# Functionally-instructed modifiers of response to ATR inhibition in experimental glioma

**DOI:** 10.1186/s13046-024-02995-z

**Published:** 2024-03-12

**Authors:** Bianca Walter, Sophie Hirsch, Laurence Kuhlburger, Aaron Stahl, Leonard Schnabel, Silas Wisser, Lara A. Haeusser, Foteini Tsiami, Sarah Plöger, Narges Aghaallaei, Advaita M Dick, Julia Skokowa, Christian Schmees, Markus Templin, Katja Schenke-Layland, Marcos Tatagiba, Sven Nahnsen, Daniel J. Merk, Ghazaleh Tabatabai

**Affiliations:** 1 Department of Neurology & Interdisciplinary Neuro-Oncology, University Hospital Tübingen, Hertie Institute for Clinical Brain Research, Eberhard Karls University Tübingen, 72076 Tübingen, Germany; 2 Cluster of Excellence (EXC 2180) “Image Guided and Functionally Instructed Tumor Therapies”, Eberhard Karls University Tübingen, 72076 Tübingen, Germany; 3 Quantitative Biology Center, Eberhard Karls University Tübingen, 72076 Tübingen, Germany; 4 Biomedical Data Science, Department of Computer Science, Eberhard Karls University Tübingen, 72076 Tübingen, Germany; 5 NMI Natural and Medical Sciences Institute, University of Tübingen, 72770 Reutlingen, Germany; 6 German Consortium for Translational Cancer Research (DKTK), Partner Site Tübingen, 72076 Tübingen, Germany; 7 Division of Translational Oncology, Department of Internal Medicine II, University Hospital Tübingen, Eberhard Karls University Tübingen, 72076 Tübingen, Germany; 8 Institute of Biomedical Engineering, Department for Medical Technologies and Regenerative Medicine, Eberhard Karls University Tübingen, 72076 Tübingen, Germany; 9 Center for Neuro-Oncology, Comprehensive Cancer Center Tübingen-Stuttgart, Eberhard Karls University Tübingen, 72076 Tübingen, Germany; 10 Present Address: Department of Neurosurgery, University Hospital Tübingen, Eberhard Karls University Tübingen, Tübingen, Germany

**Keywords:** DNA damage response pathway, Functional genomics, DigiWest, Combination therapies

## Abstract

**Background:**

The DNA damage response (DDR) is a physiological network preventing malignant transformation, e.g. by halting cell cycle progression upon DNA damage detection and promoting DNA repair. Glioblastoma are incurable primary tumors of the nervous system and DDR dysregulation contributes to acquired treatment resistance. Therefore, DDR targeting is a promising therapeutic anti-glioma strategy. Here, we investigated Ataxia telangiectasia and Rad3 related (ATR) inhibition (ATRi) and functionally-instructed combination therapies involving ATRi in experimental glioma.

**Methods:**

We used acute cytotoxicity to identify treatment efficacy as well as RNAseq and DigiWest protein profiling to characterize ATRi-induced modulations within the molecular network in glioma cells. Genome-wide CRISPR/Cas9 functional genomic screens and subsequent validation with functionally-instructed compounds and selected shRNA-based silencing were employed to discover and investigate molecular targets modifying response to ATRi in glioma cell lines in vitro, in primary cultures ex vivo and in zebrafish and murine models in vivo.

**Results:**

ATRi monotherapy displays anti-glioma efficacy in vitro and ex vivo and modulates the molecular network. We discovered molecular targets by genome-wide CRISPR/Cas9 loss-of-function and activation screens that enhance therapeutic ATRi effects. We validated selected druggable targets by a customized drug library and functional assays in vitro, ex vivo and in vivo.

**Conclusion:**

In conclusion, our study leads to the identification of novel combination therapies involving ATRi that could inform future preclinical studies and early phase clinical trials.

**Supplementary Information:**

The online version contains supplementary material available at 10.1186/s13046-024-02995-z.

## Background

The DNA damage response (DDR) is a complex network for maintaining the genetic integrity in cells [1]. This might occur, for example, by inducing cell cycle arrest upon DNA damage detection and providing cells with time to repair DNA damage [2]. Particularly the repair of three main lesions is relevant in this context: DNA double strand breaks (DSBs), DNA single strand breaks (SSB) and translesion synthesis (TLS) [3]. If the DNA damage is too severe, the DDR pathways can also steer cells towards cell death or senescence [4]. The DDR displays anti-cancer activity by halting the cell cycle upon DNA lesions and preventing mutations and cancer onset [4]. In fact, DDR genes are frequently mutated in cancers and germline mutations in DDR genes lead to hereditary cancer predisposition [5]. Furthermore, defects of DDR pathways in cancer may lead to genomic instability which in itself has been described as a hallmark of cancer [6]. Genomic instability and the concomitant replicative stress and endogenous DNA damage [7] might represent a targetable vulnerability in tumors with DDR alterations [1]. For example, poly(ADP-ribose) polymerase (PARP) inhibitors in *breast cancer gene 1* (*BRCA1*) or *BRCA2* mutated ovarian cancers exploit this cancer intrinsic vulnerability and lead to synthetic lethal interactions with clinical relevance [8, 9]. The investigation of DDR inhibitors, synthetic lethal combinations and their therapeutic potential have thus very high translational relevance in cancer research [10].

Glioblastoma are aggressive and incurable primary tumors of the central nervous system with a limited spectrum of registered therapies after maximum safe resection including radiation therapy, alkylating chemotherapy and tumor-treating fields [11, 12]. The median overall survival is still only in the range of 1.5 years, even in selected clinical trial populations [12–14]. Acquired resistance to therapy is one of the key challenges of glioblastoma treatment [12, 15]. Resistance to radiation therapy has been linked to an upregulation of DDR pathway genes, specifically the upregulation of the ataxia telangiectasia mutated (ATM)/ataxia telangiectasia and Rad3 related (ATR) pathway [16, 17]. Due to their central role in detecting DSBs and SSBs, targeting the ATR and ATM pathway has a very high translational relevance in many cancer entities [18]. The clinical application of ATR inhibitors is in early clinical development and its therapeutic challenges include bone marrow suppression after continuous AZD6738 dosing [19].

Experimental glioma overexpressing basic helix loop helix (bHLH) transcription factors display a sensitization towards ATR inhibition (ATRi) [20]. Preclinical combination therapies involving ATRi were investigated with temozolomide in O6-methylguanine-DNA methyltransferase (MGMT)-deficient glioma cells [21] as well as with the oncolytic CAN-2409/Ganciclovir system [22]. A recent phase I trial investigates the combination of ATRi with carboplatin in advanced-stage solid tumors [23].

Given the high translational relevance of combination therapies involving ATRi, our scientific objectives were (i) to discover rational combination therapies enhancing the ATRi effects in experimental glioma by genome-wide CRISPR/Cas9 drug modulator screens, transcriptomic and proteomic analysis, and (ii) to validate functionally-instructed combination strategies in vitro, ex vivo and in vivo.

## Results

### Anti-glioma activity of ATR inhibition in experimental glioma *in vitro* and *ex vivo*

ATR inhibition (ATRi) by AZD6378 and Berzosertib led to significant anti-glioma activity in human and murine glioma cell lines in vitro (Supplementary Figure S1 and S2). We confined further investigations including primary tumor cultures derived from freshly resected tumor material to one ATR inhibitor due to restricted material and restricted cell number in primary cultures (compared with long-term cell cultures). As AZD6738 has a higher ability to pass the blood-brain barrier than Berzosertib [24, 25] we preferred to use AZD6738 in these experiments. We also detected anti-tumor activity of ATRi in three patient-derived microtumors (PDM) and six primary cultures (TUE-PC1-6) ex vivo (Supplementary Figure S3). Furthermore, we observed increased induction of apoptosis upon ATRi (Supplementary Figure S4 b). Cell cycle analyses revealed an ATRi-induced accumulation in the S phase (in human LN229 and murine GL261 cells) and in the G2-M phase (LNZ308 cells) as outlined in Supplementary Figure S4d.

### ATRi-induced transcriptomic and proteomic profiles are shaped by cellular p53 status

We next selected two cell lines with diverging ATRi-induced effects on cell cycle regulation (Supplementary Figure S4d) and performed transcriptomic and proteomic analyses using RNA-sequencing (RNAseq) and DigiWest protein profiling [26] (Supplementary Figure S5 b). RNA-sequencing samples were collected in triplicates for each cell line (LN229 *n* = 3, LNZ308 *n* = 3). The principal component analysis (PCA) was primarily shaped by cell line identity rather than treatment conditions (Supplementary Figure S5a). Of note, LN229 carry mutated *TP53* and a *CDKN2A* deletion, LNZ308 cells lack *TP53* and *PTEN* [27].

**Fig. 1:**
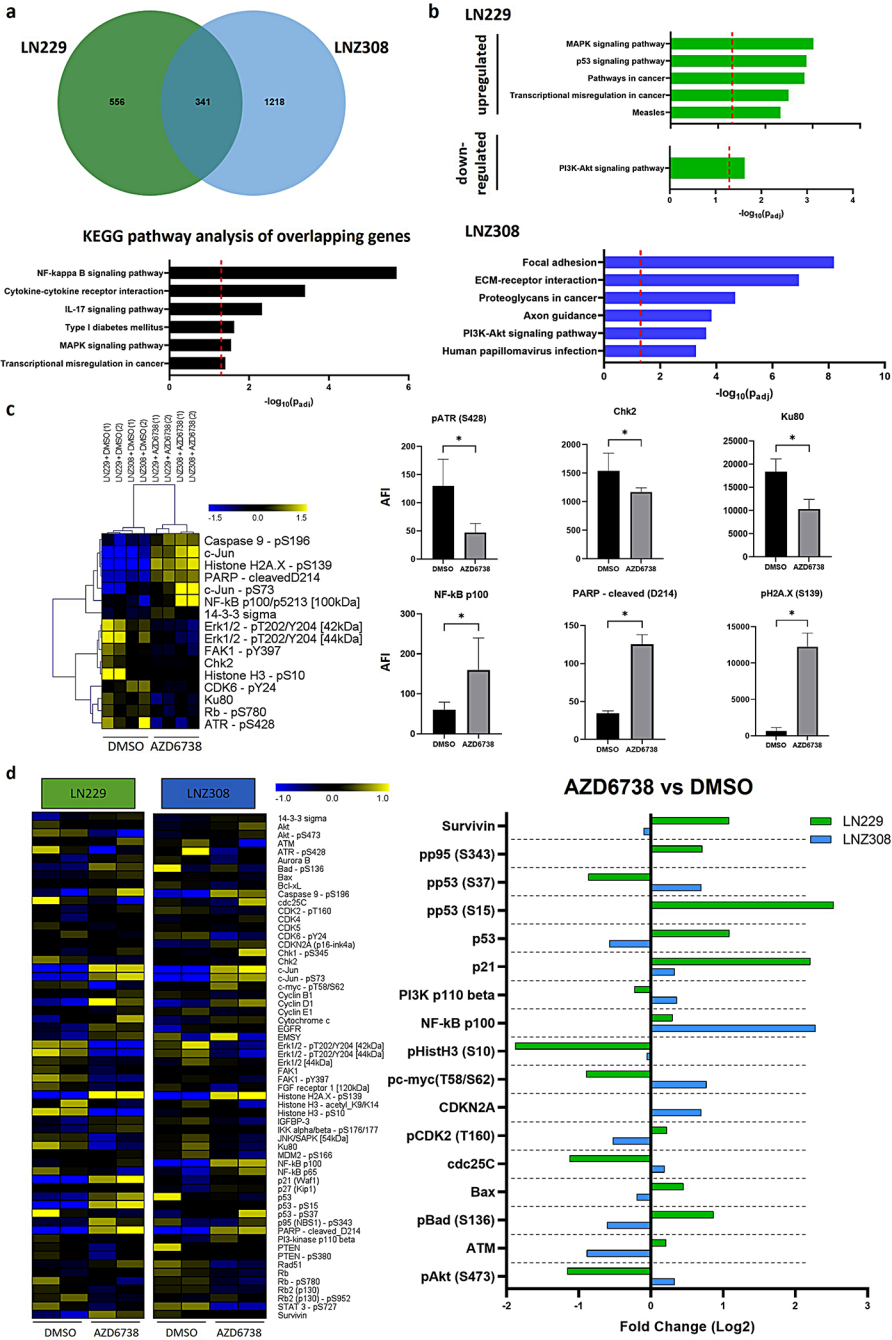
Transcriptomic and proteomic profiling before and after ATRi treatment. **a**, Venn diagram of upregulated differentially expressed genes (DEGs) in LN229 (*n* = 3) and LNZ308 (*n* = 3) cells treated with AZD6738 for 72 h. 341 upregulated genes are identified to overlap in both cell lines upon treatment. Lower panel depicts KEGG pathway analysis of identified overlapping genes. Red dashed line indicates significance level of *p* < 0.05. **b**, Based on the likelihood ratio test (LRT), genes identified to be differentially affected upon ATRi treatment in between cell lines are analyzed for KEGG pathway affiliation. p53 signaling is strongly upregulated in LN229 cells, PI3K-Akt signaling is downregulated in LN229 and upregulated in LNZ308 cells. Red dashed line indicates significance level of *p* < 0.05. **c**, DigiWest protein profiling heatmap depicting treatment- specific effects across both cell lines confirming target engagement (pATR), apoptosis induction (cleaved PARP), NFκB activation (NF-κB p100) and cell-cycle regulation (Chk2). Statistical analysis of significance for heatmap using Wilcoxon test (non-parametric, *p* < 0.05), for bar graphs Mann-Whitney test (non-parametric, rank comparison, *p* < 0.05. DMSO vs. AZD6738, LN229 *n* = 2, LNZ308 *n* = 2). **d**, Left, heatmap depicting indicated analytes separated by cell line. Right, bar graph depicting analytes differentially regulated in both cell lines upon treatment. In line with transcriptomic data, p53 is upregulated in LN229 cells while pAkt is downregulated in LN229 cells and trends towards upregulation in LNZ308 cells upon treatment

In total, we detected 1048 differentially expressed genes in LN229 cells and 2401 differentially expressed genes in LNZ308 cells upon ATRi treatment compared to respective controls (Supplementary Figure S5 b). 341 genes were upregulated (Fisher’s exact test *p* = 2.2*10^− 16^, Fig. 1a) and 22 genes were downregulated by ATRi in both cell lines (Fisher’s exact test *p* = 1.697*10^− 7^; Supplementary Figure S6). Subsequently, Kyoto encyclopedia of Genes and Genomes (KEGG) pathway analyses [28] revealed NF-κB, cytokine-cytokine receptor interaction, IL17 signaling pathway, Type I diabetes mellitus, MAPK signaling pathway and transcriptional misregulation in cancer as most altered pathways (Fig. 1a). Downregulated genes were significantly enriched for the Rap1 signaling pathway (Supplementary Figure S6).

Next, we investigated distinctly regulated genes across the two cell lines by leveraging the likelihood ratio test (LRT) and analyzed these hits again for KEGG pathway affiliation. We detected five upregulated and one downregulated pathway in LN229, and thirteen upregulated pathways in LNZ308 of which the top six are displayed (Fig. 1b). The p53 signaling pathway was significantly upregulated in LN229 cells, but not in LNZ308 cells (Fig. 1b), being in line with the p53 status of these cells [27]. Of note, PI3K-Akt signaling was regulated in an opposing manner in these glioma cell lines upon ATRi, with pathway activation in LNZ308 and inhibition in LN229 cells (Fig. 1b).

Based on these transcriptomic data, we designed a DigiWest protein profiling analysis and prepared two replicates per cell lines (LN229 *n* = 2, LNZ308 *n* = 2). The selected antibody panel (Supplementary Table ST2) covered markers for cell-cycle regulation, apoptosis, NF-κB, p53 signaling and DNA damage response.

Across both cell lines (pooled analysis of LN229 (*n* = 2) and LNZ308 (*n* = 2)), we detected a significant reduction of markers for ATR target engagement such as pATR, Chk2, and Ku80. There was a significant upregulation of NF-κB, in line with our transcriptomic data. Cleaved PARP was upregulated in both cell lines confirming the detected apoptotic response towards ATRi treatment (Fig. 1c, Supplementary Figure S4 b). Upregulation of pH2A.X in both cell lines indicated an increase in DNA damage signature (Fig. 1c). When looking at markers that were differentially regulated (Fig. 1d, separate analysis on LN229 and LNZ308 samples (*n* = 2)), LNZ308 cells display an accumulation of p16 and reduced pCDK2 (Thr160) protein levels. This signature argues for a G1 arrest [29], and our flow cytometry analyses show an accumulation of cells in G2 (Supplementary Figure S4 b). In LN229, the cell cycle regulator Survivin (BIRC5) is upregulated, CDC25C and pHistH3 (Ser10) are downregulated. Hence, for both cell lines altered regulation of the cell cycle was detected upon ATRi treatment, yet with differing underlying signal transduction pathways. Furthermore, in line with transcriptomic data, LN229 cells show an upregulation of p53, p21 and Bax and a downregulation of pAkt.

Taken together, we detected cell line specific effects of ATRi treatment on transcriptomic and proteomic levels, in particular regarding cell cycle and apoptosis regulation that indicate a specific role of the p53 signaling pathway in ATRi-mediated effects.

### Discovery of potential modifiers of response to ATRi therapy using genome-wide CRISPR/Cas9 knockout and activation screens

Next, we aimed at identifying modulators of response to ATRi in glioma cells that could further enhance the anti-glioma efficacy of ATRi by CRISPR/Cas9 functional genomic screens using genome-wide knockout (Brunello [30]) and activation sgRNA libraries (Calabrese [31]). As this experimental approach is only feasible with a high number of cells, we used two long-term cell lines for this target discovery approach.

For the discovery of potentially synthetic lethal hits by knockout screens, we first determined those ATRi concentrations that result in a cytostatic effect over the course of two weeks in both glioma cell lines (LN229 and LNZ308). For the activation screens, we then determined ATRi concentrations that result in a cytotoxic effect over the course of two weeks in both glioma cell lines (LN229 and LNZ308) to identify potential modifiers of treatment response, accordingly.

**Fig. 2:**
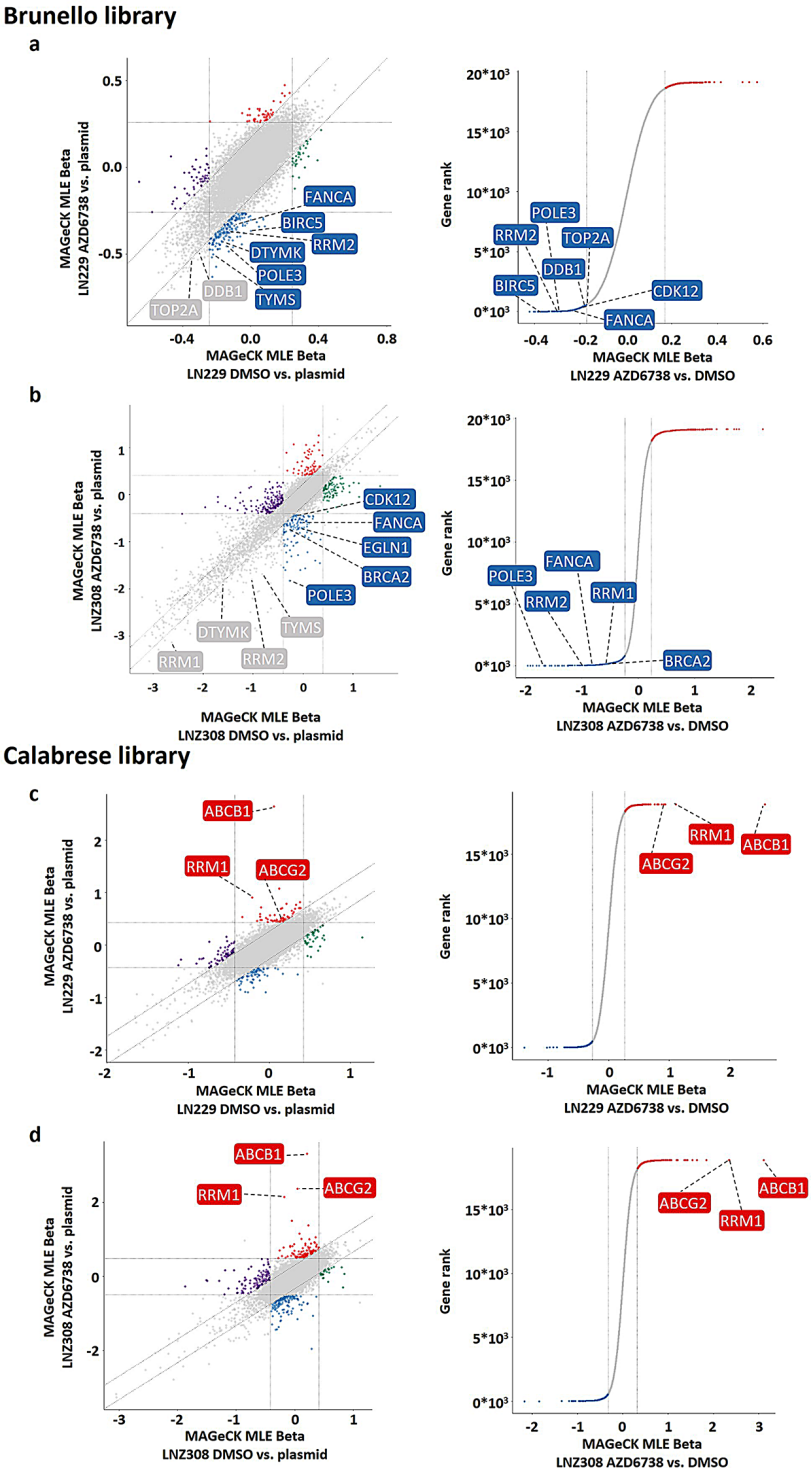
CRISPR-Cas9 genome-wide knockout and activation screens identify novel combination partners for ATRi. **a, b** Upper half, CRISPR-Cas9 screen analyses using a knockout (Brunello) library in LN229 (**a**) and LNZ308 cells (**b**) using 750 nM of AZD6738. Left, 9-square plot of MAGeCK MLE results comparing Brunello sgRNA distributions from DMSO or AZD6738-treated cells to the plasmid library pool. Right, rankview plot illustrating MAGeCK MLE results comparing Brunello sgRNA distributions from AZD6738-treated cells to the corresponding DMSO control. **c, d** Lower half, CRISPR-Cas9 screen analyses using an activation (Calabrese) library in LN229 (**c**) or LNZ308 cells (**d**), respectively, using 1.5 µM AZD6738 for LN229 and 1.4 µM AZD6738 for LNZ308 cells. Left, 9-square plot of MAGeCK MLE results comparing Calabrese sgRNA distributions from DMSO or AZD6738-treated cells to the plasmid library pool. Right, rankview plot illustrating MAGeCK MLE results comparing Calabrese sgRNA distributions from AZD6738-treated cells to the corresponding DMSO control. The respective experimental set-up was used to prioritize genetic vulnerabilities (Brunello library) and resistance mechanisms (Calabrese library) upon ATR inhibition

We observed a depletion of known pan-essential genes in both knockout screens, indicating good screen performance (Supplementary Figure S7 c). We defined potential synthetic lethal genetic vulnerabilities as depleted hits in the treatment condition while unchanged in the DMSO condition (Supplementary Figure S7 b, left, blue population). Based on this, we compiled a list of treatment-related genetic vulnerabilities for each screen. Hits in DNA damage repair associated genes such as *FANCA* and *BRCA2* (Fig. 2a, b), further confirmed the validity of the screens. By comparing the hits of both cell lines, we determined overlapping and distinct hits.

We defined potential modifiers of treatment response after gene activation as enriched genetic signatures in the treatment condition while being unchanged in the DMSO condition (Supplementary Figure S7 b, right, red population). Interestingly, the most prominent hits in both cell lines were *ABCB1*, *ABCG2* and *RRM1* (Fig. 2c, d). The ATP binding cassette (ABC) transporters have been reported to confer resistance to chemotherapy in several cancer entities [32]. The finding of *RRM1* as treatment-related enrichment in the activation setting complements the finding of the knockout screens where *RRM1* and *RRM2* knockout led to depletion under treatment (Fig. 2a, b).

### Functionally-instructed drug screens reveal cisplatin, fludarabine phosphate and hydroxyurea as most promising combination candidates for ATRi

**Fig. 3:**
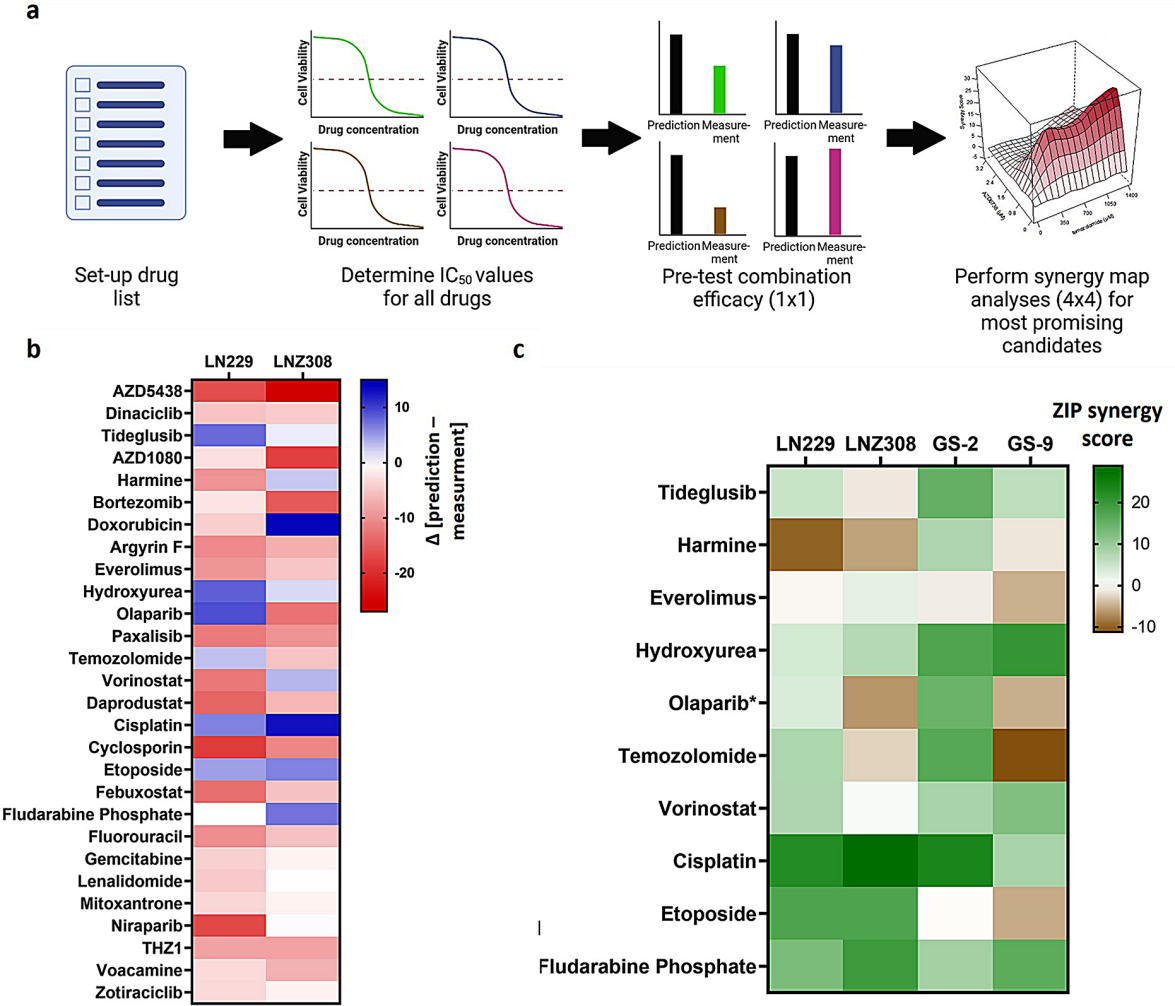
Functionally-instructed combination therapies *in vitro*. **a**, Schematic workflow of the functionally-instructed drug screen. For all selected drugs IC_50_ values were determined. Then, a pre-selection screen, i.e. 1 × 1 (IC_50_xIC_50_) combination of AZD6738 plus drug of interest, was set-up. All combinations with AZD6738 resulting in higher efficacy than additive interaction were then included in 4 × 4 synergy map analyses. **b**, Analysis of the 1 × 1 pre-selection screen (*n* = 1 with 8 technical replicates per sample). The heatmap depicts the delta value between prediction of additive drug-drug interaction and measured viability (Bliss synergy score). Positive values (blue) indicate a higher efficacy of the drug combination than predicted, negative values (red) indicate a lower efficacy of the drug combination than predicted. Tideglusib, harmine, everolimus, hydroxyurea, olaparib, temozolomide, vorinostat, cisplatin, etoposide and fludarabine phosphate were selected as top candidates. **c**, Analysis of the 4 × 4 synergy map experiments. Heatmap depicts the average ZIP synergy score across tested combinations (*n* = 2). Green coloring indicates high synergism scores, brown coloring negative synergism scores. Hydroxyurea, cisplatin and fludarabine phosphate show positive synergism values across all four cell lines tested

We compared all identified hits with the drug gene interaction database (DGIdb). Upon further considerations regarding druggability, we also investigated vulnerabilities that were not strictly within our borders, e.g., *TOP2A*, *DDB1* (Fig. 2a). We created a drug library of 28 compounds (Supplementary Table ST3) for further validation of functionally-instructed targets as potential combination partners for ATRi in experimental glioma (Fig. 3a). We then performed cytotoxicity assays to investigate synergistic effects. In the pre-test screen, we determined Bliss synergy scores in LN229 and LNZ308 cells for all 28 compounds combined with AZD6738 drug treatment. We identified tideglusib, harmine, doxorubicin, hydroxyurea, olaparib, temozolomide, vorinostat, cisplatin, etoposide and fludarabine phosphate as top scoring hits (Fig. 3b). The subsequent 4 × 4 synergy map analyses were performed in the two long term cell lines LN229 and LNZ308 as well as in the glioma stem-like cells GS-2 and GS-9. All pre-selected drugs displayed a synergistic signature in at least one of the cell lines (Fig. 3c, exemplary heatmap read-out for GS-2 cells treated with hydroxyurea can be found in Supplementary Figure S8).

The combination of ATRi plus olaparib or temozolomide, respectively, displayed an interesting synergism pattern. We detected a synergistic interaction in both p53 expressing cells (LN229 and GS-2) and a lack of synergistic interaction in both p53 null cell lines (LNZ308 and GS-9) for these combinations (Fig. 3c, Supplementary Figure S 9 a, S10 a). We reasoned that the p53 status might be relevant in this regard. Indeed, silencing of p53 in LN229 and in GS-2 cells (Supplementary Figure S9 b, S10 b) diminished the synergy of ATRi in combination with olaparib (Supplementary Figure S9 c, d) indicating a determining role of p53 expression for a combination treatment of ATRi plus olaparib.

In the context of ATRi plus temozolomide combination treatment, the MGMT status had been previously defined as a predictive factor for synergistic combination of ATRi plus temozolomide in glioma cells [21]. However, GS-2 cells have intact MGMT protein (Supplementary Figure S 9 a, S10 a) and ATRi plus temozolomide still leads to synergistic treatment effect in these cells (Fig. 3c). Yet, silencing of p53 diminished the synergistic effect of temozolomide and ATRi, particularly in GS-2 cells (synergy score 9.3 in GS-2 shLuciferase cells and 0.7 in GS-2 shTP53 cells, Supplementary Figure S9 e). LN229 cells, on the other hand do not have MGMT protein (Supplementary Figure S9 a, S10 a), and ATRi plus temozolomide leads to synergistic treatment effects in these cells, too, but p53 silencing has only a modest effect on the synergistic interaction of ATRi plus temozolomide in LN229. We concluded that p53 expression is relevant for synergistic interaction of ATRi plus temozolomide, particularly in MGMT-expressing glioma cells.

Furthermore, we detected a consistent synergistic signature of ATRi in combination therapies involving Hydroxyurea, cisplatin and fludarabine phosphate in all four cell lines and irrespective of p53 status and MGMT status (Fig. 3c). Consequently, we selected these three combination treatments for subsequent ex vivo and in vivo experiments.

### Synergistic anti-glioma effects of ATRi combined with cisplatin or fludarabine phosphate *ex vivo* and *in vivo*

**Fig. 4:**
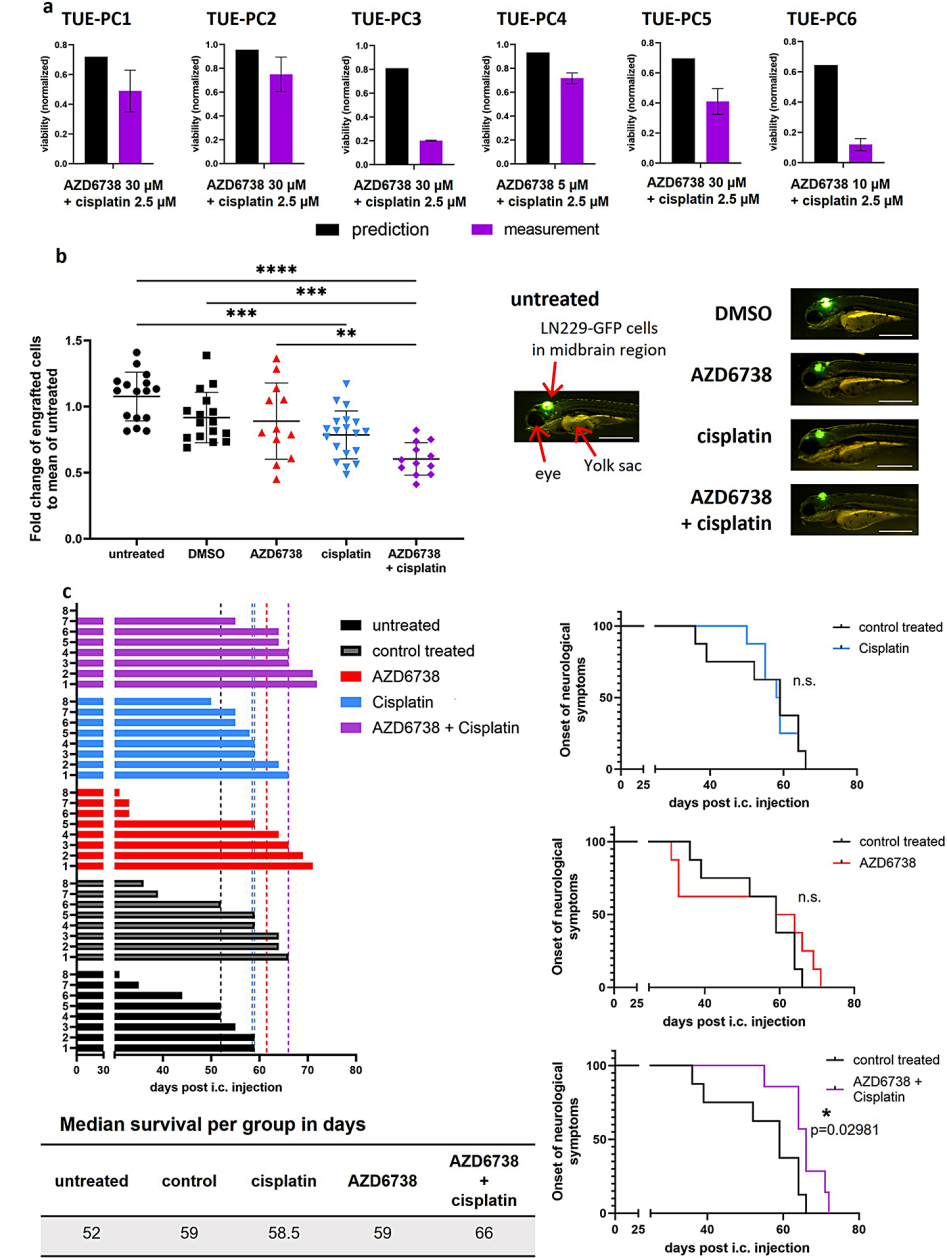
Combination of AZD6738 with Cisplatin or Fludarabine Phosphate show synergistic efficacy *ex vivo* and *in vivo*. **a**, Evaluation of combination treatment efficacy and synergy of AZD6738 with cisplatin in primary glioma cultures (PC). Shown as black bar is the predicted value for additive combination effects based on the Bliss Independence Criterion as outlined in Methods. Observed measurements (purple) of combination treatments are depicted in purple. Lower values than predicted indicate a synergistic effect of the combination. Shown are means ± SD (*n* = 1, 3 technical replicates per sample). Representative bar graphs for one AZD6738 treatment in combination with cisplatin in each PC. **b**, Tumor areas of control (untreated (*n* = 16), DMSO (*n* = 16)), AZD6738 [50 µM] (*n* = 12), cisplatin [150 µM] (*n* = 19) and the combination of both (*n* = 12) treated wildtype zebrafish embryos xenotransplanted with LN229-GFP cells. Data was collected in two independent experiments. Tumor surface areas are measured using Imaris (version 9.2.0) after 48 h of treatment. Measurements were normalized to untreated control, means ± SD of the respective groups are indicated, each dot represents one embryo. Statistical analysis using one-way ANOVA (all to all comparison of means), Sidak correction for multiple testing, shown are only comparisons with corrected p-values < 0.5. * *p* < 0.05, ** *p* < 0.01, *** *p* < 0.001, **** *p* < 0.0001. Right panel, exemplary pictures of zebrafish embryos of each group. Left, untreated zebrafish embryo with anatomical features “eye”, “yolk sac” and “LN229-GFP cells in midbrain region” highlighted by red arrows. Scale bars: 500 μm. **c**, Waterfall graph (left) and Kaplan-Meier curves (right) of untreated, control treated, AZD6738 (50 mg/kg), cisplatin (1 mg/kg), AZD6738/cisplatin-treated nude mice transplanted with LN229 cells. Median survival for each group listed below waterfall plot. Statistical analysis using log-rank (Mantel Cox) test, p-value below 0.05 considered significant, n.s. “not significant”. Combination of AZD6738 plus cisplatin significantly prolongs survival compared to control treated mice

We next validated selected functionally-instructed combination therapies (i.e., combination efficacy of ATRi plus cisplatin, fludarabine phosphate or hydroxyurea) in six different primary cultures TUE-PC1-6 (Supplementary Figure ST4). Cisplatin and fludarabine phosphate led to synergistic read-outs in all six primary cultures (Fig. 4a, Supplementary Figure S11, S12). Hydroxyurea led to a synergistic readout in TUE-PC2, TUE-PC3 and TUE-PC6 but not in TUE-PC1, TUE-PC4, TUE-PC5 (Supplementary Figure S13).

We selected the combination of ATRi and cisplatin for further validation in zebrafish embryos in vivo. Both monotherapies only led to a modest decrease of tumor area, which was not significant as compared to the DMSO control (Fig. 4b). The combination of ATRi and cisplatin was significantly more efficacious. In a murine model, ATRi and cisplatin significantly prolonged survival in vivo compared with controls (median control: 59 d, median combination: 66 d, *p* = 0.02981) (Fig. 4c).

## Discussion

DDR genes are frequently mutated in several cancer entities, including glioblastoma [10]. This often results in a dependency of cancer cells towards the remaining DDR pathways and an actionable vulnerability [8, 33]. A well-established clinical example in this regard is the use of PARP inhibitors in *BRCA1* or *BRCA2* mutated ovarian cancers [9, 34].

Acquired resistance to therapy with subsequent tumor progression is a major challenge in the treatment of glioblastoma. Current treatment guidelines in glioblastoma include radiation therapy and the alkylating drugs temozolomide and lomustine [13, 14, 35]. These alkylating drugs predominantly lead to DNA damage by single strand breaks [36, 37] and ATR activation. Furthermore, high ATR expression is associated with poor survival in glioblastoma patients [38], and ATRi leads to reduced invasion of glioblastoma cells through dysregulated cytoskeletal networks [38]. Thus, ATRi is a promising therapeutic strategy for glioma and could be leveraged to exploit treatment-induced vulnerabilities.

ATRi monotherapy leads to anti-glioma efficacy in vitro and ex vivo (Supplementary Figure S1-3) as has been reported previously [20–22]. Mechanistically, we observed distinct ATRi-induced molecular alterations in glioma cells and cell cycle alterations connected to p53 signaling and depending on cellular p53 status (Figs. 1 and 3, Supplementary Figure S 9, S10). For example, LN229 cells upregulate p21 and downregulate p-Histone H3, indicative of cell cycle control through p53 signaling [39, 40], which is not detectable in LNZ308 cells (Fig. 1). In acute cytotoxicity assays, LN229 and GS-2 cells display a synergistic interaction of ATRi with temozolomide or olaparib, which is not detectable in LNZ308 or GS-9 cells (Fig. 3c). LN229 and GS-2 cells have measurable p53 protein while LNZ308 and GS-9 do not [27, 41] and silencing of p53 reverted these effects (Supplementary Figure S9, S10). ATRi in combination with olaparib overcomes PARPi resistance in breast cancer and in ovarian cancer models [42], and this combination is already included in treatment arms of clinical trials like OLAPCO (NCT02576444) and CAPRI (NCT03462342). Our data add an additional layer to these findings and advocate for considering the p53 status for this combination.

A role of *MGMT* gene promoter methylation (and lack of MGMT) for a synergistic efficacy of ATRi plus temozolomide has been described before [21]. In our study, silencing of p53 strongly modifies the synergistic effect of ATRi and temozolomide in GS-2 cells (Supplementary Figure S 9). GS-2 cells display intact MGMT protein (Supplementary Figure S9a, S10) and display a synergistic interaction of ATRi with temozolomide (Fig. 3c) which is diminished upon p53 silencing. In the absence of MGMT, however, silencing of p53 does not significantly modify the synergistic efficacy of ATRi and temozolomide, as outlined in LN229 cells (Supplementary Figure S 9 c). Our data thus indicate a role for p53 signaling when considering ATRi plus temozolomide in MGMT-unmethylated glioblastoma and advocate for considering the p53 status in this context.

Using genome-wide CRISPR/Cas9 activation screens during treatment with ATRi, we identified the activation of ABC transporters, i.e., *ABCB1* and *ABCG2*, in both cell lines (Fig. 2c, d). Based on this, we selected voacamine, a cannabinoid (CB) antagonist [43] and ABCB1 inhibitor [44], and cyclosporin for the drug library. Yet, simultaneous combination of ATRi plus ABC transporter inhibition did not lead to a synergistic cytotoxic read-out (Fig. 3b). Potentially, the sequential treatment of voacamine and ATRi might be a future candidate approach. Leveraging the CRISPR/Cas9 knockout library, we discovered a number of different genetic hits that could instruct synthetic lethal combination therapies. Many of the detected hits were associated with DDR or DNA replication, e.g., *FANCA*, *BRCA2, DNA damage-binding protein 1 (DDB1)*, *DNA polymerase epsilon (POLE3), ribonucleotide reductase catalytic subunit M1 (RRM1)* (Fig. 2a, b). Following these functionally-instructed molecular targets, we aimed to induce genotoxic stress and thereby overpower the other DDR pathways. A similar approach has been conducted in experimental glioma and ovarian cancer using PARP1 inhibition plus temozolomide and WEE1 inhibition plus carboplatin, respectively [45, 46]. Taking these results into account, we included cisplatin, fludarabine phosphate and etoposide in the drug library for chemical validation. Additionally, we included direct inhibitors of genetic hits associated with DDR and DNA replication like hydroxyurea (*RRM1*) [47] or lenalidomide (*DDB1*) [48]. Cisplatin, hydroxyurea and fludarabine phosphate led to a synergistic interaction in all four tested glioma cell lines (Fig. 3c), in turn, validating the efficacy of our workflow. We also investigated these combinations in primary cultures ex vivo (Fig. 4a, Supplementary Figure S11-13). We observed a synergistic read-out in all six tested primary cultures for cisplatin and fludarabine phosphate and in three using hydroxyurea in combination with ATRi.

Cisplatin (or derivatives) together with ATRi, which showed robust synergistic outcomes ex vivo (Fig. 4a), have been studied in other contexts before. Pre-clinical data in breast cancer xenograft mouse models showed favorable outcomes of ATRi in combination with carboplatin (among others) [49]. In another study, cisplatin resistance of head and neck squamous cell carcinoma could be overcome in vitro and in vivo by adding VE-822, a p-ATR inhibitor [50]. Furthermore, in a Phase 1 clinical trial of berzosertib together with cisplatin (NCT02157792) in advanced solid tumors (but not glioblastoma) preliminary clinical activity of this combination was detected [51]. In vitro data using LY294002, an inhibitor of the DNA DSB detector DNA-dependent protein kinase (DNA-PKcs) [52], in combination with cisplatin increased cytotoxicity in the glioma cell line U343 [53]. Based on our data and these previous observations, we investigated the ATRi/cisplatin combination in zebrafish embryos orthotopically xenotransplanted with glioma cells in vivo and detected a significantly stronger reduction of tumor area in ATRi plus cisplatin-treated zebrafish embryos compared to untreated or DMSO-treated embryos (Fig. 4b). Furthermore, simultaneous ATRi/cisplatin significantly improved survival in an orthotopic murine glioma model (Fig. 4c).

## Conclusion

Taken together, our study incorporating “static” transcriptomic and proteomic analysis (RNAseq and DigiWest) in concert with dynamic genome-wide CRISPR/Cas9 functional genomics led to the discovery of combination approaches involving ATRi in experimental glioma. To balance the necessity of long-term cell lines for genome wide functional genomics, we validated here selected functionally-instructed combination therapies in several preclinical models in vitro, ex vivo and in vivo. Additionally, we provide data for a novel role of the p53 status in glioma cell lines as a determining factor for synergistic effects of ATRi plus olaparib as well as ATRi plus temozolomide in the absence of MGMT. Since DDR dysfunction, and particularly ATRi, is a relevant strategy in several diseases, our data will be interesting beyond the field of neuro-oncology and can provide a resource for promising further modifiers of response to ATRi.

## Methods

### Long term cell lines and primary glioblastoma cell cultures

We cultured LN229, LNZ308, GL-261 and SMA560 glioma cell lines in DMEM (Gibco, Carlsbad, CA, US) supplemented with 10% fetal calf serum (Thermo Fisher Scientific, Waltham, MA, US) and 50 µg/mL Gentamycin (Thermo Fisher Scientific, Waltham, MA, US). GS-2 and GS-9 were cultured in Neurobasal®-A Medium (Gibco, Carlsbad, CA, US) supplemented with 2% B27 without vitamin A (Invitrogen, Waltham, MA, US), 1% GlutaMAX (Thermo Fisher Scientific, Waltham, MA, US), 0.02 µg/mL fibroblast growth factor (FGF) and epidermal growth factor (EGF) (PeproTech, Cranbury, NJ, US) and 50 µg/mL Gentamycin (Thermo Fisher Scientific, Waltham, MA, US). Standard culture conditions are 5% CO_2_ at 37 °C.

Primary tumor tissue was obtained from fresh residual material upon resection at the Department of Neurosurgery, University Hospital Tübingen. We cut the tissue in small pieces, washed it using Hanks Balanced Salt Solution (HBSS) (Gibco, Carlsbad, CA, US) and digested it by collagenase and dispase (Roche, Basel, CH). To remove any remaining erythrocytes, we used red blood cell lysis buffer (Sigma Aldrich, St. Louis, MO, US). We then cultured the cells as GS cells in Neurobasal®-A medium. We first evaluated ATRi sensitivity of each primary culture and selected the two concentrations closest to IC25 for further experiments. These two concentrations were then combined with two concentrations of Cisplatin (1 and 2.5 µM), Fludarabine Phosphate (10 and 20 µM) and Hydroxyurea (150 and 200 µM). To evaluate combination treatment analyses, we leveraged the Bliss Independence Criterion as described in the drug screen method section.

### Patient derived microtumors (PDM)

PDMs were extracted from surgically resected, residual glioblastoma tissue as has been published before [54–56]. In brief, the tissue was minced into small pieces and necrotic tissue discarded. We washed the remaining tissue in Hank’s Balanced Salt Solution (HBSS, Thermo Fisher, Waltham, MA, US) and digested the tissue using Liberase DH (Sigma Aldrich, St. Louis, MO, US). We washed the tissue again and sequentially filtered the samples, removing any single cells. Remaining PDMs were collected from the top of the cell strainer and cultured in 60 mm dishes containing StemPro hESC SFM medium (Thermo Fisher, Waltham, MA, US) with addition of bFGF (10 µg mL − 1; Peprotech, Rocky Hill, NJ, US) and 1% Primocin (Invivogen, San Diego, CA, US) at 5% CO2 and 37 °C in a humidified incubator. The previous studies also include histological characterization of the generated PDMs [54–56].

We treated the PDMs with indicated drug concentrations for 72 h and determined cell viability using RealTime-Glo MT Cell Viability Assay (Promega, Madison, WI, US). Luminescence assay signal was measured using a multimode microplate reader (Tecan, Männedorf, Switzerland). Obtained luminescence units were background corrected and plotted as dose response curves using GraphPad Prism 9 software. The use of residual tissue after tumor resections was approved by the ethical board of the University Hospital Tübingen.

### Functionally-instructed compound library

As ATRi, we used AZD6738 (Celasertib, Selleckchem, Houston, TX, US) at a stock concentration of 50 mM in dimethylsulfoxide (DMSO) and Berzosertib (ChemiTek, Indianapolis, IN, US) also diluted at 50 mM in DMSO as ATR inhibitors. Furthermore, we generated a compound library based on the functionally-instructed targets for drug screens. All compounds are outlined in Supplementary Table ST3.

### Acute cytotoxicity assay

As described [54], we seeded 5 000 or 10 000 cells, respectively, on day one, treated the next day in serum-free medium and incubated for up to 72 h. Subsequently, we measured cell viability using CellTiterBlue reagent (Promega, Madison, WI, US) according to manufacturer’s instructions with a GloMax (Promega, Madison, WI, US). We normalized measurements to untreated cells.

### Clonogenic survival assay

As previously published, cells were seeded the day before treatment [54]. We treated the cells in the indicated concentrations in serum-free medium for 24 h after which we changed the medium back to medium containing FCS, followed by a 7 to 21 day incubation time. We used crystal violet (0.5% w/v) to stain colonies and analyzed the area coverage using the ImageJ software PlugIn ColonyArea as described in the publication by Guzman et al. [57]. We normalized measurements to respective vehicle control wells.

### Annexin V/PI – flow cytometry analysis

For this, the FITC Annexin V Apoptosis Detection Kit I (Beckton, Dickinson & Company, Franklin Lakes, NJ, US) was used according to manufacturer’s protocol. In brief, we seeded the cells on day one and treated them on the next day. 48 h after treatment, we detached the cells, stained and analyzed them within 1 h on a MACSQuant Analyzer 10 (Miltenyi Biotec, Bergisch Gladbach, Germany). Next, we analyzed the acquired data with the FlowJo software (Beckton, Dickinson & Company, Franklin Lakes, NJ, US). The gating strategy is outlined in Supplementary Figure S5.

### Cell cycle analysis

We conducted this method as described before [54]. In brief, we seeded the cells on day one and treated them on the next day. After 72 h, we detached the cells and stained them with propidium iodide solution (50 µg/mL propidium iodide (Thermo Fisher Scientific, Waltham, MA, US), 0,2% Triton X-100 (Roth, Karlsruhe, Germany), 100 µg RNase (Thermo Fisher Scientific, Waltham, MA, US), 1 g/L glucose in PBS (phosphate buffered saline) (Gibco, Carlsbad, CA, US). Flow cytometry was done using a MACSQuant Analyzer 10 (Miltenyi Biotec, Bergisch Gladbach, Germany). We analyzed the acquired data with the FlowJo software (Beckton, Dickinson & Company, Franklin Lakes, NJ, US). The gating strategy is outlined in Supplementary Figure S6.

### RNA-Sequencing (RNAseq)

For this, we seeded the cells on day one and treated them on the next day with IC_50_ concentrations of AZD6738 for up to 72 h in triplicates (*n* = 3). We extracted the RNA using the Qiagen RNeasy Mini kit (Qiagen, Venlo, NL) according to manufacturer’s protocol. For RNA sequencing, we enriched the mRNA fraction using polyA capture from 200 ng of total RNA using the NEBNext Poly(A) mRNA Magnetic Isolation Module (NEB). We used the NEB Next Ultra II Directional RNA Library Prep Kit for Illumina (NEB) to prepare mRNA libraries according to manufacturer’s instructions. The libraries were sequenced as paired-end 50 bp reads on an Illumina NovaSeq6000 (Illumina) with a sequencing depth of approximately 25 million clusters per sample. We performed RNA raw data QC and processing using megSAP (version 0.2-135-gd002274) combined with ngs-bits package (version 2019_11-42-gflb98e63). To align the reads to the GRCh38 we used STAR v2.7.3a [58] and alignment quality was analyzed using ngs-bits. Normalized read counts for all genes were obtained using Subread (v2.0.0) and edgeR (v3.26.6). We used the R package “DESeq2” [59] for further analyses. We defined differentially expressed genes (DEGs) as log2 fold-change (LFC) above or below |1| and p-adjusted < 0.01 as defined by Wald statistics. We analyzed the resulting hits for KEGG pathway association using gProfiler [60]. We also used the likelihood ratio test (LRT) which is included in the “DESeq2” package to identify significantly differentially changed genes between treated cell lines. We re-aligned significant LRT genes with DEG results and analyzed them with gProfiler [60].

### DigiWest multiplex protein analysis

After 72 h of AZD6738 treatment, we detached and counted the cells and subsequently snap froze them in liquid nitrogen. For protein preparation, we kept the cells on ice and added 20–30 µL of lysis buffer (LDS Lysis Buffer (Life Technologies, Carlsbad, CA, USA), supplemented with reducing agent (Thermo Fisher Scientific, Waltham, MA, USA) and Protease- (Roche Diagnostics GmbH, Mannheim, Germany) and Phosphatase-Inhibitor (Roche)). Proteins were denatured by heating to 95 °C for 10 min before the lysates were transferred to QiaShredder Eppendorf tubes (Eppendorf, Hamburg, GER). After centrifugation (16,000 g, 5 min, RT), eluates were stored at -80 °C until further use. Protein quantification was performed using in-gel staining. 1 µL of each original lysate was diluted 1:10 (v/v) in lysis buffer. The respective aliquots were denatured for 10 min at 70 °C and 10 µL were run in a NuPAGE 4–12% Bis-Tris precast gel (Thermo Fisher Scientific) according to the manufacturer´s instructions. The gel was washed with water and proteins were stained with BlueBandit (VWR, Darmstadt, GER) for 1 h. The gel was de-stained over night with ddH_2_O before detection on a LI-COR (LI-COR, Bad Homburg, GER) instrument. Analysis and protein quantification was performed using ImageStudio.

The DigiWest analysis was performed as published previously [26]. In brief, we loaded 10 µg of cellular protein on an SDS-polyacrylamide gel and size-separated them using the commercial NuPAGE system (Life Technologies). Size-separated proteins were blotted onto a PVDF membrane and biotinylated on the membrane using NHS-PEG12-Biotin (50 µM) in PBST for 1 h. After drying of the membrane, the samples lanes were cut into 96 strips of 0.5 mm width using an automated cutting plotter (Silhouette America, West Orem, UT, US) each corresponding to a defined molecular weight fraction. Each of the strips was placed in one well of a 96-well plate and 10 µl elution buffer (8 M urea, 1% Triton-X100 in 100 mM Tris-HCl pH 9.5) was added. The eluted proteins were diluted with 90 µl of dilution buffer (5% BSA in PBS, 0.02% sodium azide, 0.05% Tween-20) and each of the protein fractions was incubated with 1 distinct magnetic color-coded bead population (Luminex, Austin, US) coated with neutravidin. The biotinylated proteins bind to the neutravidin beads such that each bead color represents proteins of one specific molecular weight fraction. All 96 protein loaded bead populations were mixed resulting in reconstitution of the original lane. Such a bead-mix was sufficient for about 150 individual antibody incubations (Supplementary Table ST2). Aliquots of the DigiWest bead-mixes (about 1/200th per well) were added to 96 well plates containing 50 µl assay buffer (Blocking Reagent for ELISA (Roche, Rotkreuz, CH) supplemented with 0.2% milk powder, 0.05% Tween-20 and 0.02% sodium azide) and different diluted antibodies were added to the wells. After overnight incubation at 15 °C in a shaker, the bead-mixes were washed twice with PBST and species-specific PE-labelled (Phycoerythrin) secondary antibodies (Dianova, Hamburg, GER) were added and incubated for 1 h at 23 °C. Beads were washed twice prior to readout on a Luminex FlexMAP 3D.

For quantification of the antibody specific signals, we used the DigiWest Analyzer software; it automatically identifies peaks of appropriate molecular weight and calculates the peak area. Signal intensity was normalized to the total amount of protein loaded onto one lane. The software package MEV 4.9.0 was used for statistical analysis [61] along with Graph Pad Prism (Version 9.0.0). For all statistical tests, a p-value < 0.05 was considered significant.

### CRISPR/Cas9 genome-wide knockout screens

The genome-wide CRISPR/Cas9 knockout library Brunello was previously described and targets 19,114 human genes with 76,441 sgRNAs [30]. For generation of Cas9 expressing cells, glioma cell lines were transduced with a lentivirus coding for Cas9 (gift from Feng Zhang (Addgene plasmid #52,962; http://n2t.net/addgene:52962; RRID:Addgene_52962)) [62] in the presence of polybrene (4 or 8 µg ml^− 1^, respectively). Following transduction, we transduced cells and selected them with a predetermined concentration of blasticidin for five days, and verified Cas9 expression using immuno blots.

For genome-wide knockout screens, we transduced a total of 225*10^6 (LN229) and 200*10^6 (LNZ308) Cas9 expressing cells with a predetermined appropriate volume of lentiviral-packaged Brunello library to achieve a maximum 30% transduction efficiency maintaining a 500x library coverage. We performed transductions in technical duplicates. 24 h after transduction, cells were selected with a predetermined concentration of puromycin for a total of five days, and transduction efficacy was evaluated using an in-line assay. Starting on day 7 of the screen, for each duplicate we split 40*10^6 cells to drug or corresponding DMSO arms, thereby maintaining a coverage of 500x. We regularly split all screen arms under continuous drug or vehicle treatment for a total of 14 days, maintaining a minimum library coverage of 500x at all time. We collected 60*10^6 cells, pelleted and stored them at -80 °C for DNA extraction.

### CRISPR Cas9 genome-wide activation screens

Similarly to knockout screens outlined above, we used CRISPR screens leveraging the previously described Calabrese P65-HSF library (Set A) for genome-wide gene activation [31]. This library targets a total of 18,885 gene promoters with a set of 56,762 sgRNAs. Glioma cell lines LN229 and LNZ308 used for activation screens were transduced with lentiviral particles to stably express dCas9-VP64 prior to screening according to the protocol outlined above (gift from Feng Zhang (Addgene plasmid #61,425; http://n2t.net/addgene:61425; RRID:Addgene_61425)) [63].

For genome-wide activation screens, we transduced a total of 450*10^6 (LN229) and 180*10^6 (LNZ308) dCas9-VP64 expressing cells with a predetermined appropriate volume of lentiviral-packaged Calabrese library to achieve a maximum 30% transduction efficiency maintaining a 500x library coverage. We performed transductions in technical duplicates. 24 h after transduction, we selected cells with a predetermined concentration of puromycin for a total of five days, and transduction efficacy was evaluated using an in-line assay. Starting on day 7 of the screen, for each duplicate we split 30*10^6 cells to drug or corresponding DMSO arms, thereby maintaining a coverage of 500x. We regularly split all screen arms under continuous drug or vehicle treatment for a total of 14 days. In DMSO control arms, a minimum library coverage of 500x was kept at all times. Due to cytotoxic drug concentrations, drug arms fell under the 500x coverage early during the screen, and all surviving cells were kept throughout the entire screen. For DMSO control arms 50*10^6 cells, for treatment conditions all remaining cells were collected, pelleted and stored at -80 °C for DNA extraction.

### CRISPR Cas9 screen analysis

We extracted the DNA from screen pellets using the QIAmp DNA Blood Kit (Qiagen, Venlo, NL) according to manufacturer’s protocol. We sent the resulting DNA to the Broad Institute of MIT and Harvard for next generation sequencing using a previously described protocol [30]. Amounts of DNA subjected to sequencing were estimated to provide a 500x library coverage, assuming 6.6 pg of genomic DNA per eukaryotic cell. Additionally, sequencing data from the corresponding plasmid pools of Brunello and Calabrese libraries used to generate the lentiviral particles used in this study were provided by the Broad Institute. After next generation sequencing (NGS) by the Broad Institute, quality control assessments were done using FastQC (v0.11.9). Reads were mapped to the corresponding sgRNA libraries and counted using PoolQ (3.4.3). PoolQ processed in average a total of 1.6 million reads, with an average mapping rate of 75.5%. Each sgRNA instance was counted and a tab delimited count file was generated. We used a custom script (available at https://github.com/LaurenceKuhl/poolQcrisprcleanR) for file and table formatting. Log_2_ fold changes from both drug and DMSO arm replicates compared to the plasmid reference and corresponding reads from CRISPR/Cas9 knockout studies were corrected for gene independent effects using crisprcleanR (v3.0.1). Then, corrected reads counts were further analyzed using the MAGeCK MLE algorithm with the MAGeCK software (0.5.9.5) [64] to identify screen hits. MAGeCK MLE utilizes a maximum-likelihood estimation (MLE) for robust identification of CRISPR-screen hits and assigns beta scores and corresponding FDR statistics to assess gene depletion or enrichment in the screens. Screens were compared to either the corresponding DMSO controls or the plasmid reference as indicated by the design matrices used for MAGeCK MLE. Screen visualization was generated with MAGeCKFlute (3.16). Genes further considered all presented an FDR value below 10% when compared with plasmid or DMSO.

### Functionally-instructed design of a compound library for the drug screens

We leveraged an acute cytotoxicity assay workflow to evaluate potential interaction partners for ATRi which were derived from transcriptomic, proteomic and genome-wide CRISPR/Cas9 genetic dependency analyses. In a 1 × 1/IC_50_xIC_50_ set-up, we treated LN229 and LNZ308 cells in serum free medium for 72 h. We used the original Bliss Independence Criterion to evaluate synergism potential. For this, a predicted value for additivity, derived from the product of the two monotherapy settings, was compared to the actual measurement of the combined treatment of the two drugs. Lower values than predicted point towards synergy, higher values towards antagonism [65]. Subsequently, we subjected LN229, LNZ308, GS-2 and GS-9 cells to 4 × 4 synergy map assays again in an acute cytotoxicity assay workflow. We used the R package synergyfinder with zero interaction potential (ZIP) synergy scores calculated from 4 × 4 concentration matrices for each tested combination and the average across the plate [66]. Averages were used to produce an overview heatmap of the drug screen and evaluate the best combination approach.

### Generation of TP53 knockdown cells

The cloning strategy followed the Addgene cloning protocol provided for the pLKO.1-TRC cloning vector [67]. Small changes included the annealing strategy, we heated the Oligos to 95 °C for 5 min and afterwards cooled them down from 90 °C decreasing the temperature by 5 °C every minute until reaching 25 °C. The pLKO.1 puro vector was digested with AgeI and EcoRI in one step. We produced lentiviral particles using HEK293FT cells. We transfected LN229 and GS-2 cells with the respective shLuciferase and shTP53 virus and selected with puromycin. Successful knockdown was evaluated by immunoblot.

### Immunoblot analyses

We performed immunoblot analyses as published previously [20, 54]. In brief, we lysed the cells using RIPA buffer (Merck KGaA, Darmstadt, Germany). We used 10% polyacrylamide gels and transferred the proteins onto a polyvinylidene fluoride (PVDF) membrane (Thermo Fisher Scientific, Waltham, MA, US). Blots were blocked in Tris-buffered saline containing 5% skim milk (Beckton, Dickinson & Company, Franklin Lakes, NJ, US). Primary antibodies were applied over night at 4 °C, secondary antibodies for 1 h at room temperature. We detected the results documented with a ChemiDoc MP imaging system (Bio-Rad, Hercules, CA, US). Subsequent analysis was performed with Image Lab software (Bio-Rad, Hercules, CA, US). We used the p53 antibody Sc-263 (Santa Cruz, Dallas, TX, US), MGMT ab39253 (Abcam, Cambridge, UK) and GAPDH 2118 (Cell Signaling, Technology, Danvers, MA, US). As secondary antibodies we used goat pAb to mouse IgG (HRP), ab97023 (Abcam, Cambridge, UK) and goat pAb to rabbit IgG (HRP) ab97051 (Abcam, Cambridge, UK).

### Orthotopic glioma model in zebrafish

We used zebrafish wildtype (wt) TE embryos younger than 5 days post-fertilization (dpf). Zebrafish lines were kept according to standard protocols and handled in accordance with European Union animal protection directive 2010/63/EU and approved by the local government (Tierschutzgesetz § 11, Abs. 1, Nr.1, husbandry permit 35/9185.46/Uni TÜ). We stably transduced cells with pLJM1-EGFP (Addgene plasmid #19,319; http://n2t.net/addgene:19319; RRID:Addgene_19319) [68]. Labelled cells were suspended in PBS at 2*10^5^ cells/µL and approx. 1 nL cell suspension was injected into the midbrain region of 24 hpf embryos. We incubated the embryos at 28 °C for 1 h and afterwards divided them into treatment groups. Only successfully transplanted embryos were used for further experiments. Then, we applied the treatments as indicated in the respective figures and incubated that for 48 h at 35 °C. For evaluation of tumor size, we imaged embryos on a Nikon Stereomicroscope (SMZ18) with the NIS Element software. We assessed the tumor surface areas using Imaris (version 9.2.0).

### Orthotopic xenograft glioma mouse model

All animal experiments were approved by the regional council Tübingen and conducted in accordance with animal law. Animals used in this study were ordered from Charles River Germany and kept in the animal facility of the institute. They are regularly analyzed for infectious diseases. We injected 75,000 LN229 glioma cells into the right striatum of female nude NU/NU CD1 mice (Charles River, Sulzfeld, GER) as described [20, 54, 56]. On day 7 post surgery, we randomized mice into treatment (AZD6738, Cisplatin, combination) and control (untreated, vehicle) arms. We administered AZD6738 (Celasertib, Selleckchem, Houston, TX, US) or vehicle control via oral gavage at 50 mg/kg bodyweight for five days followed by two days of treatment holiday for four weeks. We used a concentration of 1 mg/kg bodyweight for cisplatin (Medchemexpress, Monmouth Junction, NJ, US) treatment and administered it two times a week for two weeks. Animals were closely monitored as shown in the scoring sheets (Supplementary Table ST1). The endpoint of the experiment was according to the animal law “the time until the onset of neurological symptoms”, in the text designated as “survival”.

### Statistical analysis

For statistical analyses we used log-rank (Mantel-Cox) test, multiple unpaired t tests, Mann-Whitney test, one-way ANOVA, Fisher’s exact test as suitable and indicated in the respective figure legends. All replicates are derived from distinct samples and Gaussian distribution of all samples was assumed. We assumed significance when adjusted p-values were below 0.05, shown are mean ± SD or median values and all normalizations are indicated in the figure legends. For animal experiments, we performed a biostatistical assessment. For sample size planning we aimed at a power of 80%, assuming normal distribution and standard deviation based on previous experiments for the time until onset of neurological symptoms.

*In LN229 AZD6738/Olaparib analyses the highest AZD6738 concentration was removed from the calculation of median synergy scores. Exemplary heatmap for GS-2 cells treated with hydroxyurea plus AZD6738 can be found in Supplementary Figure S8.

### Electronic supplementary material

Below is the link to the electronic supplementary material.

Supplementary Material 1

Supplementary Material 2

Supplementary Material 3

Supplementary Material 4

Supplementary Material 5

Supplementary Material 6

Supplementary Material 7

Supplementary Material 8

Supplementary Material 9

Supplementary Material 10

Supplementary Material 11

Supplementary Material 12

Supplementary Material 13

Supplementary Material 14

## Data Availability

The datsets and computer code produced in this study are available in the following databases: •RNASeq data: Gene Expression Omnibus GSE229614 (https://www.ncbi.nlm.nih.gov/geo/query/acc.cgi?acc=GSE229614). This data will be publicly available upon publishing. For the peer review process, we provide a token via e-mail to the editorial office. •For file and table formatting of CRISPR/Cas9 screens, we used a custom script that is available at https://github.com/LaurenceKuhl/poolQcrisprcleanR.

## Abbreviations

ATR: ataxia telangiectasia and Rad3 related
ATRi: ATR inhibition
ATM: ataxia telangiectasia mutated
bHLH: basic helix loop helix
DDR: DNA damage response
DGIdb: drug gene interaction data base
DSB: DNA double strand break
KEGG: Kyoto encyclopedia of genes and genomes
LRT: likelihood ratio test
PC: primary culture
PDM: patient derived microtumor
RNAseq: RNA sequencing
SSB: DNA single strand break
TLS: translesion synthesis

## Declarations

### Patent filing

Parts of this scientific publication are the subject of (non-published) European patent application EP 23 177 739.2.
