# Supplementary figures and images for: Functionally-instructed modifiers of response to ATR inhibition in experimental glioma
Source: J Exp Clin Cancer Res. 2024 Mar 12;43:77. doi: 10.1186/s13046-024-02995-z (PMC10935927; doi:10.1186/s13046-024-02995-z)

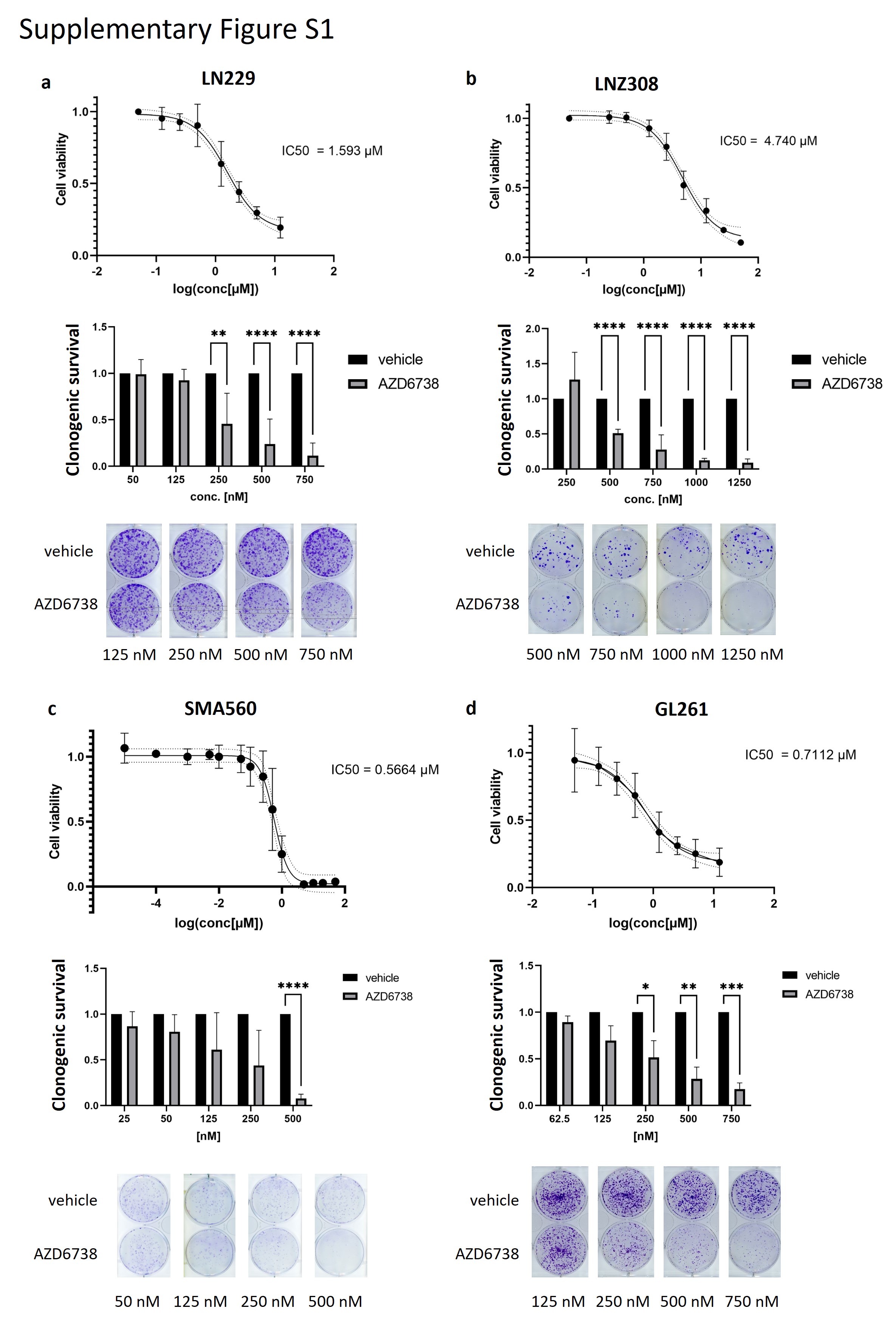

Supplement: Supplementary file 1 — Supplementary Material 1 [file 13046_2024_2995_MOESM1_ESM.jpg]

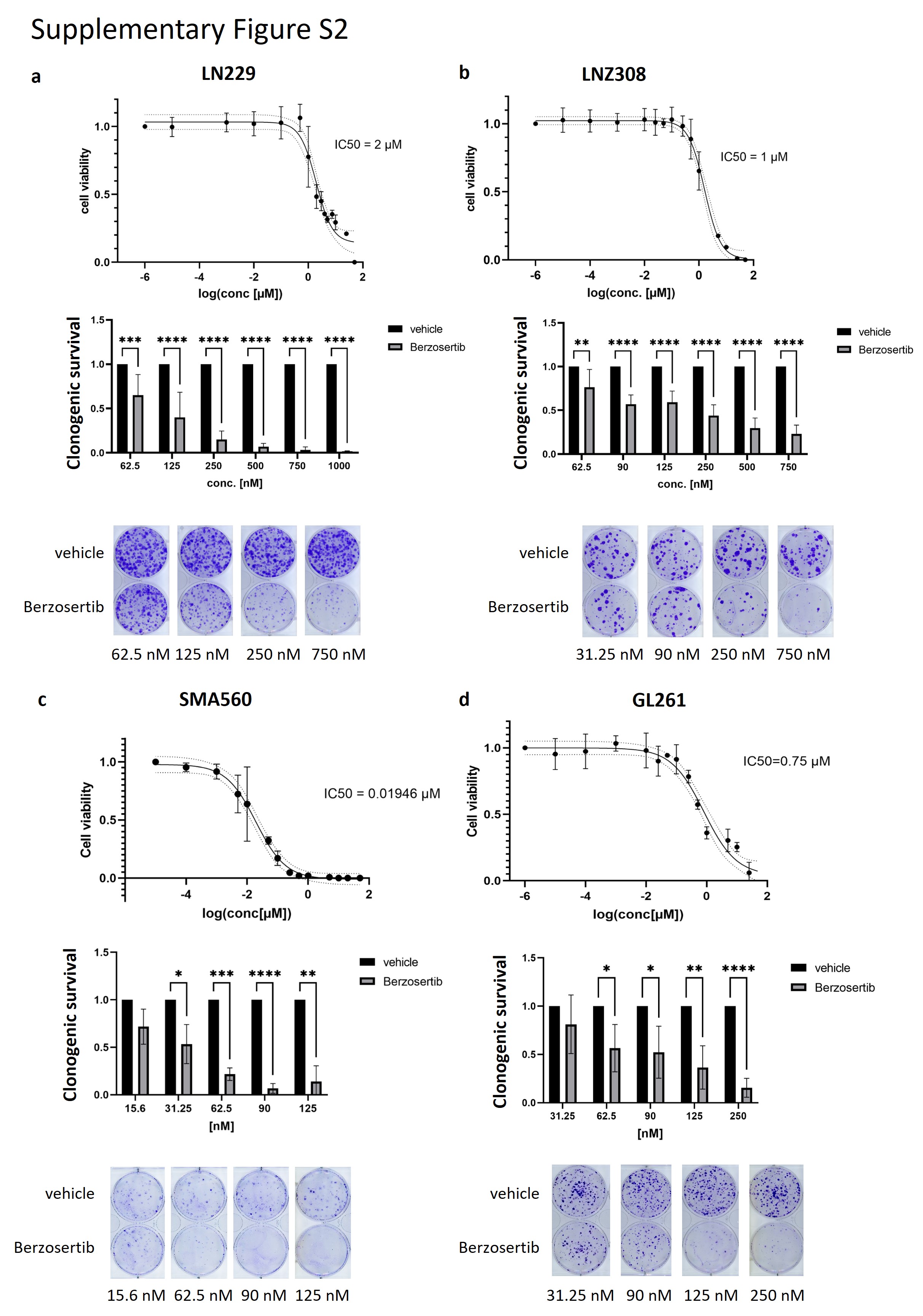

Supplement: Supplementary file 2 — Supplementary Material 2 [file 13046_2024_2995_MOESM2_ESM.jpg]

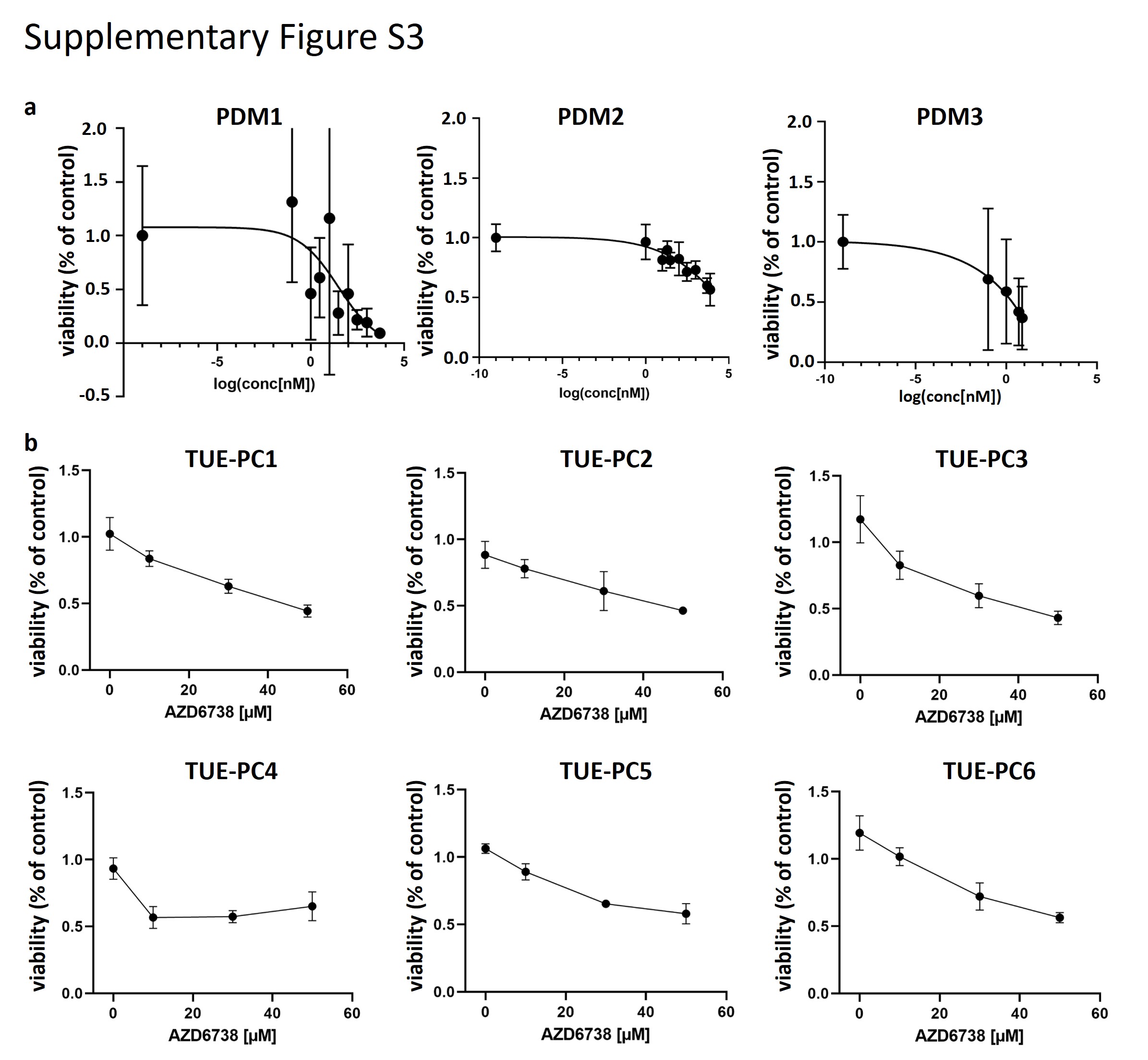

Supplement: Supplementary file 3 — Supplementary Material 3 [file 13046_2024_2995_MOESM3_ESM.jpg]

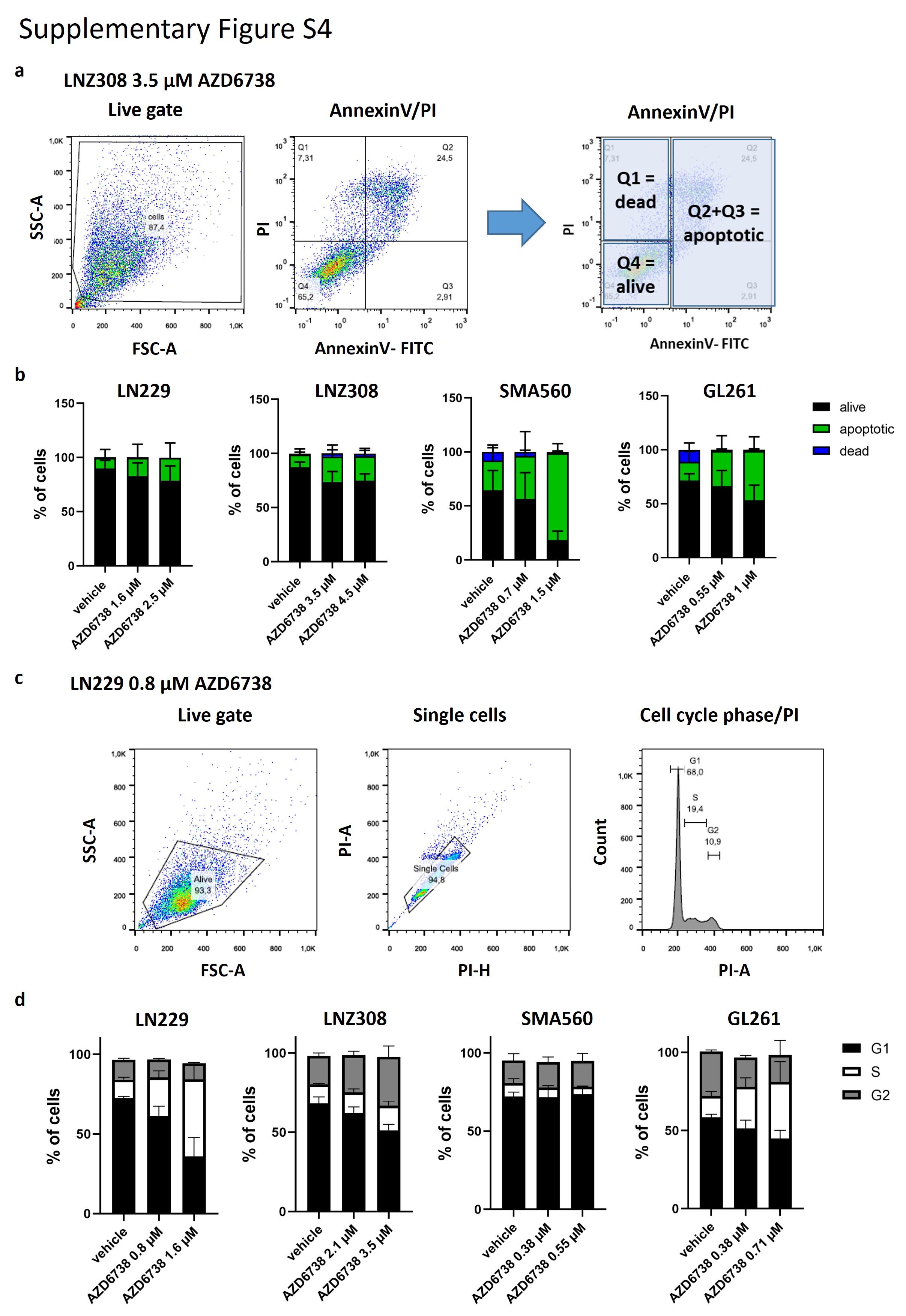

Supplement: Supplementary file 4 — Supplementary Material 4 [file 13046_2024_2995_MOESM4_ESM.jpg]

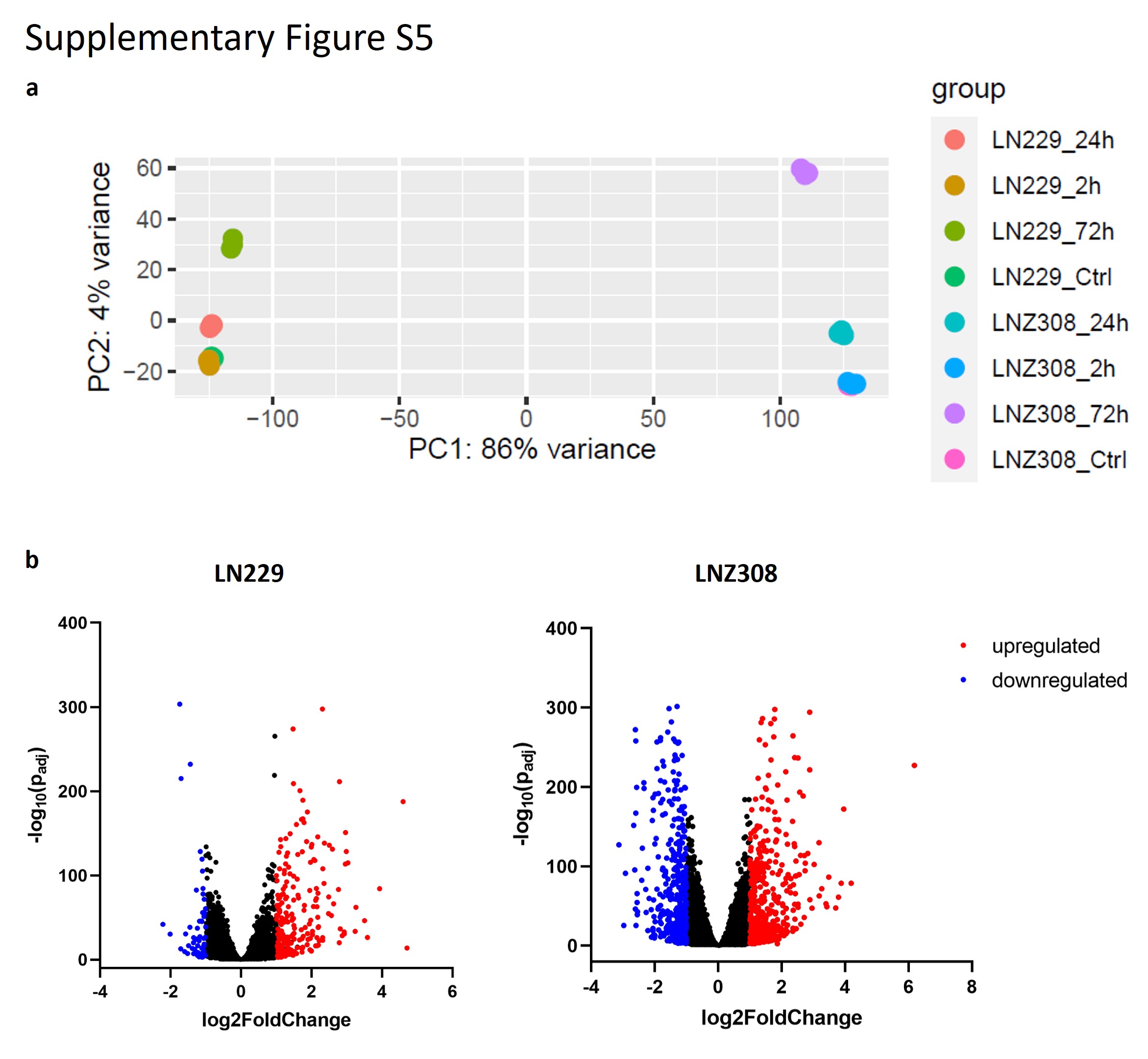

Supplement: Supplementary file 5 — Supplementary Material 5 [file 13046_2024_2995_MOESM5_ESM.jpg]

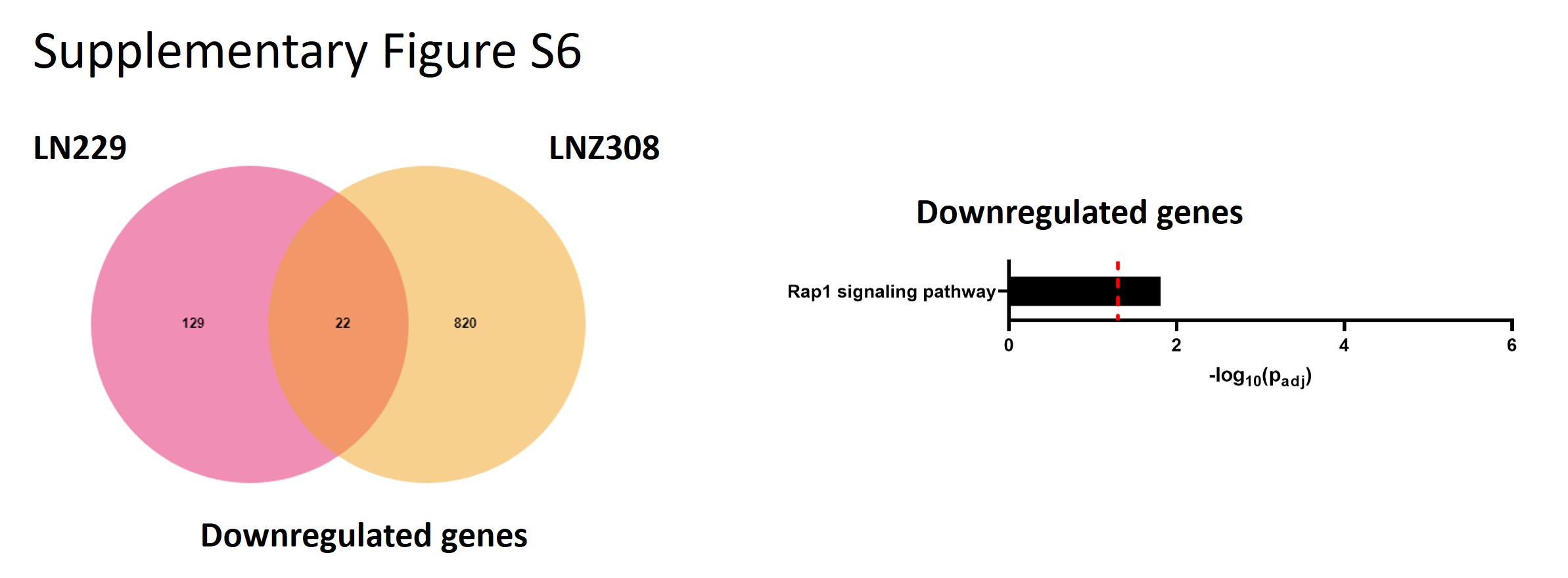

Supplement: Supplementary file 6 — Supplementary Material 6 [file 13046_2024_2995_MOESM6_ESM.jpg]

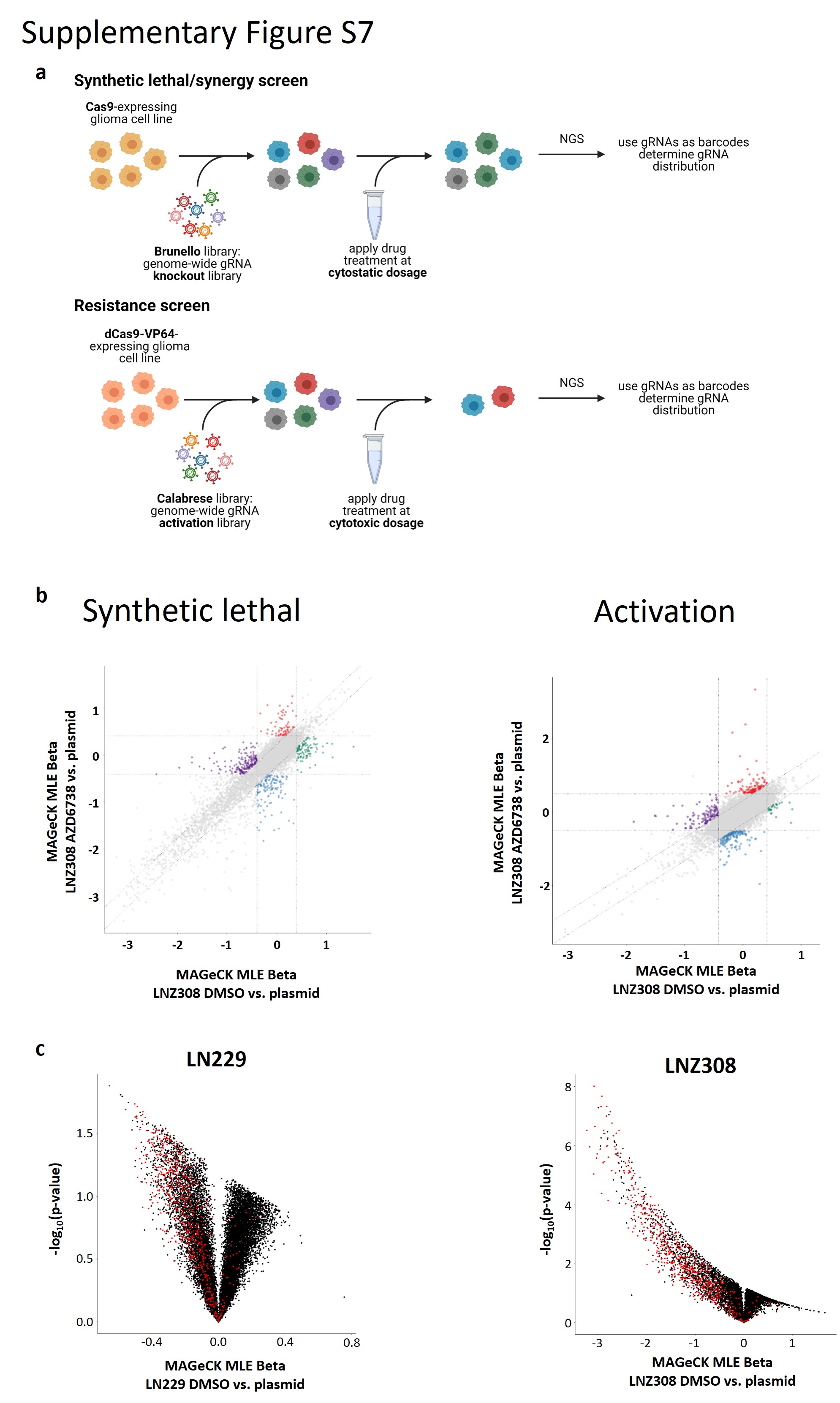

Supplement: Supplementary file 7 — Supplementary Material 7 [file 13046_2024_2995_MOESM7_ESM.jpg]

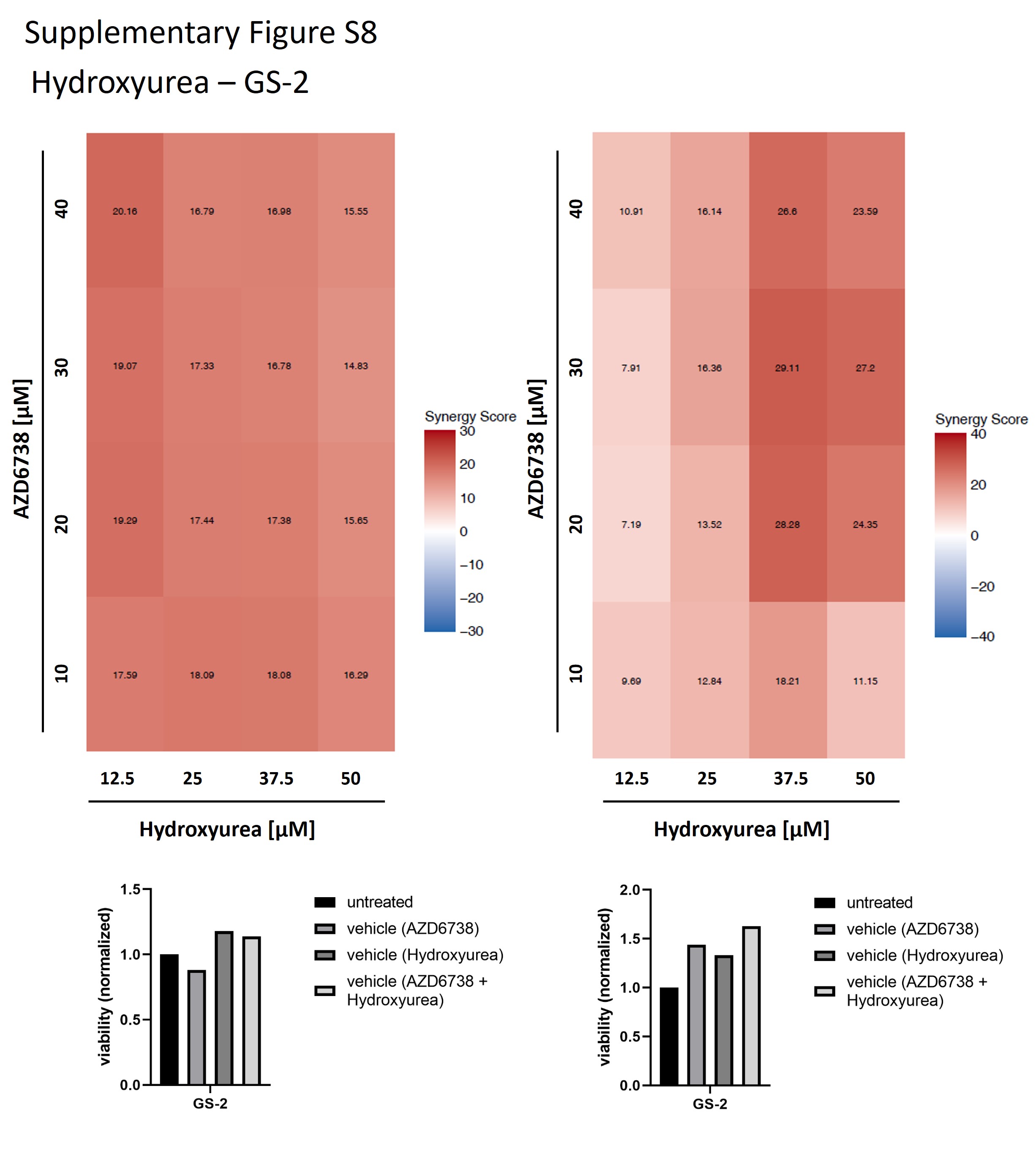

Supplement: Supplementary file 8 — Supplementary Material 8 [file 13046_2024_2995_MOESM8_ESM.jpg]

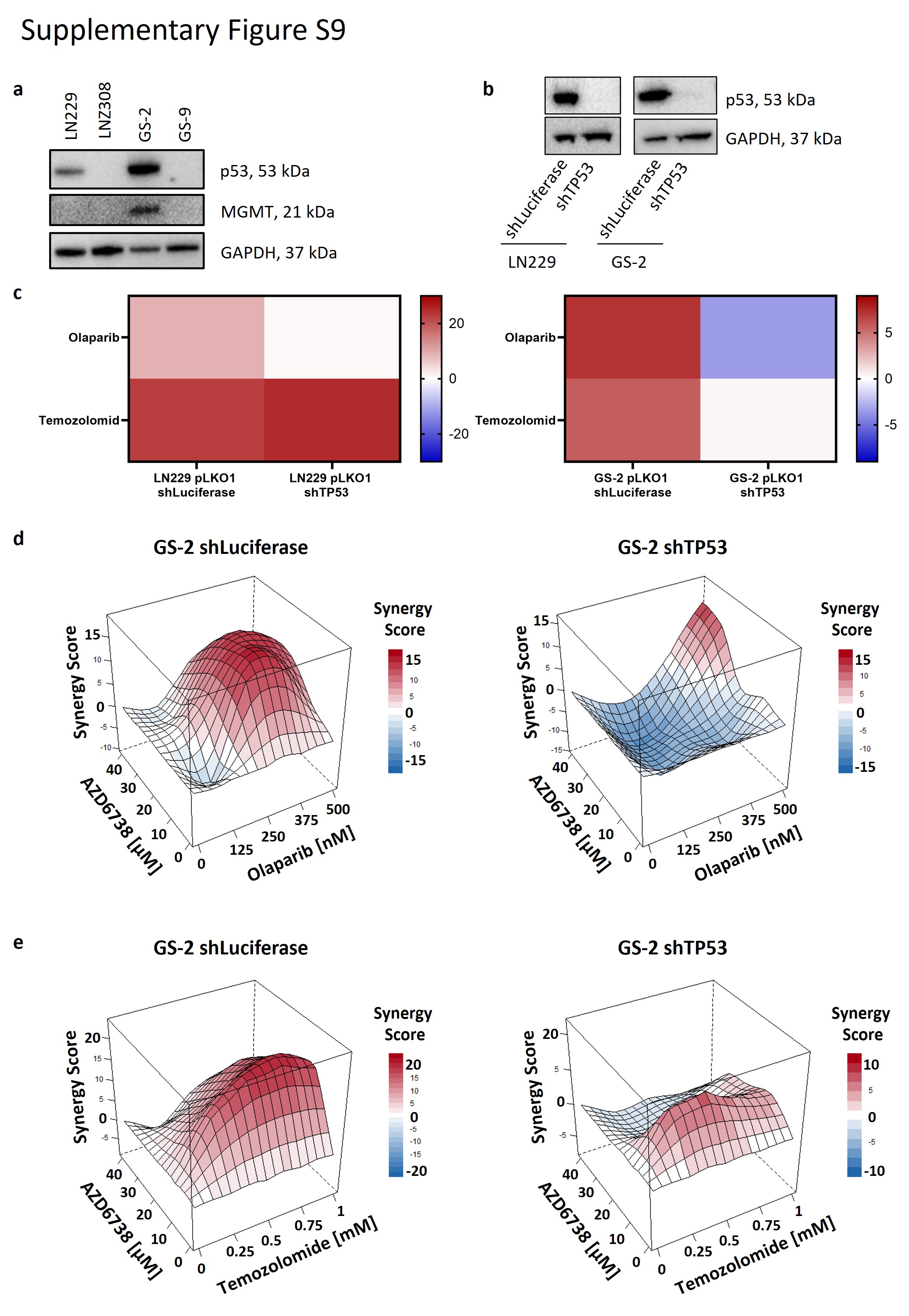

Supplement: Supplementary file 9 — Supplementary Material 9 [file 13046_2024_2995_MOESM9_ESM.jpg]

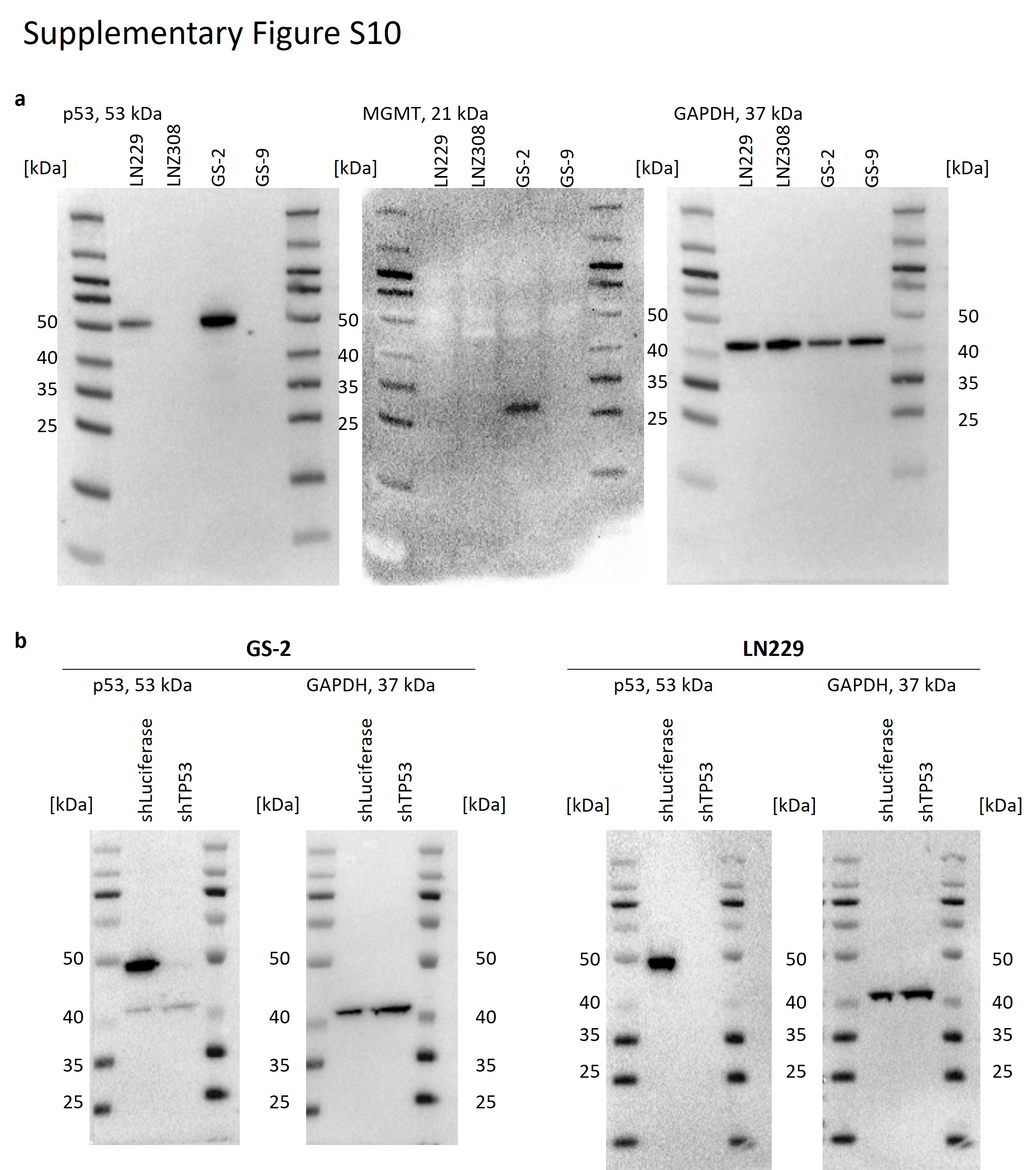

Supplement: Supplementary file 10 — Supplementary Material 10 [file 13046_2024_2995_MOESM10_ESM.jpg]

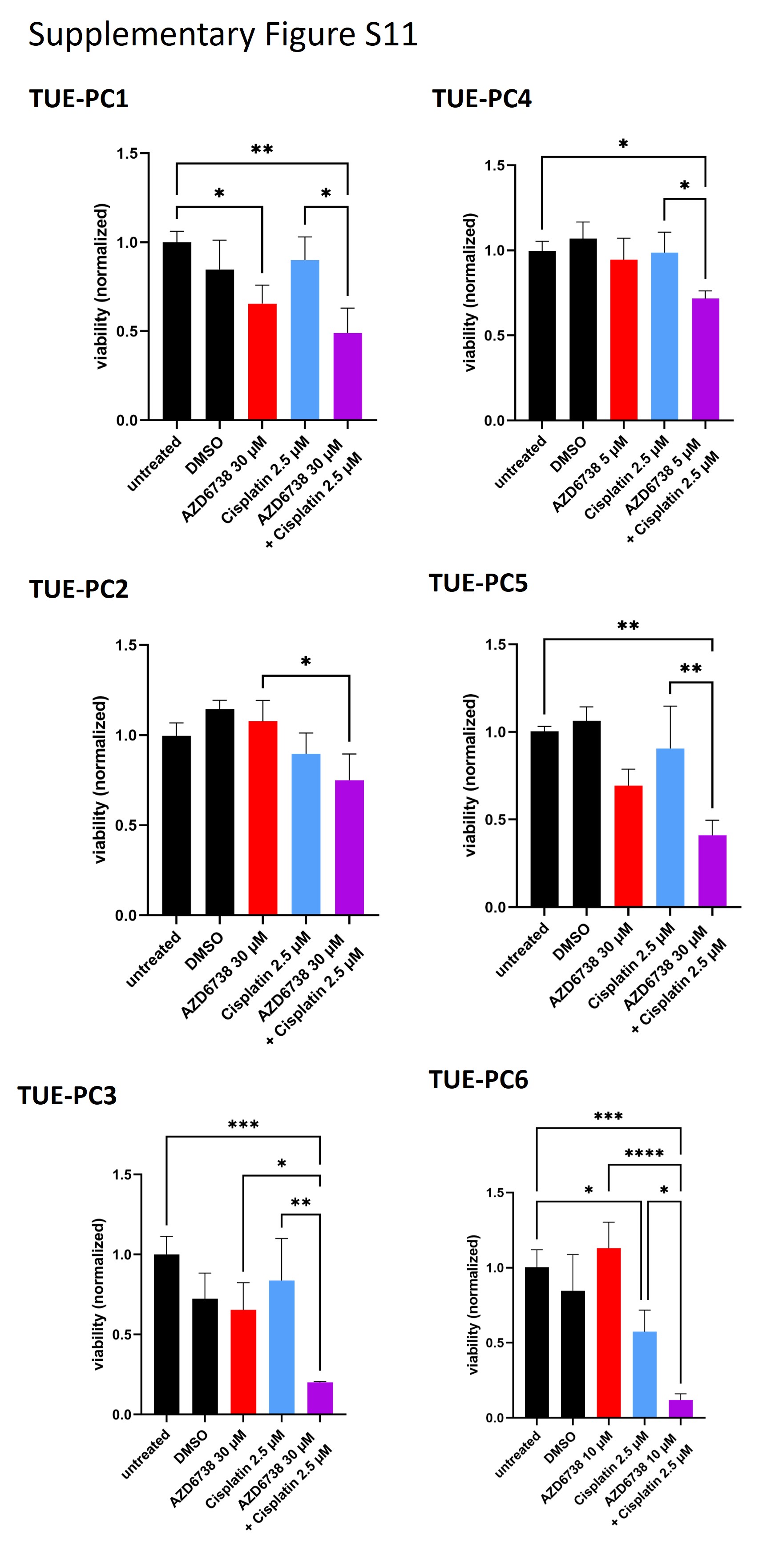

Supplement: Supplementary file 11 — Supplementary Material 11 [file 13046_2024_2995_MOESM11_ESM.jpg]

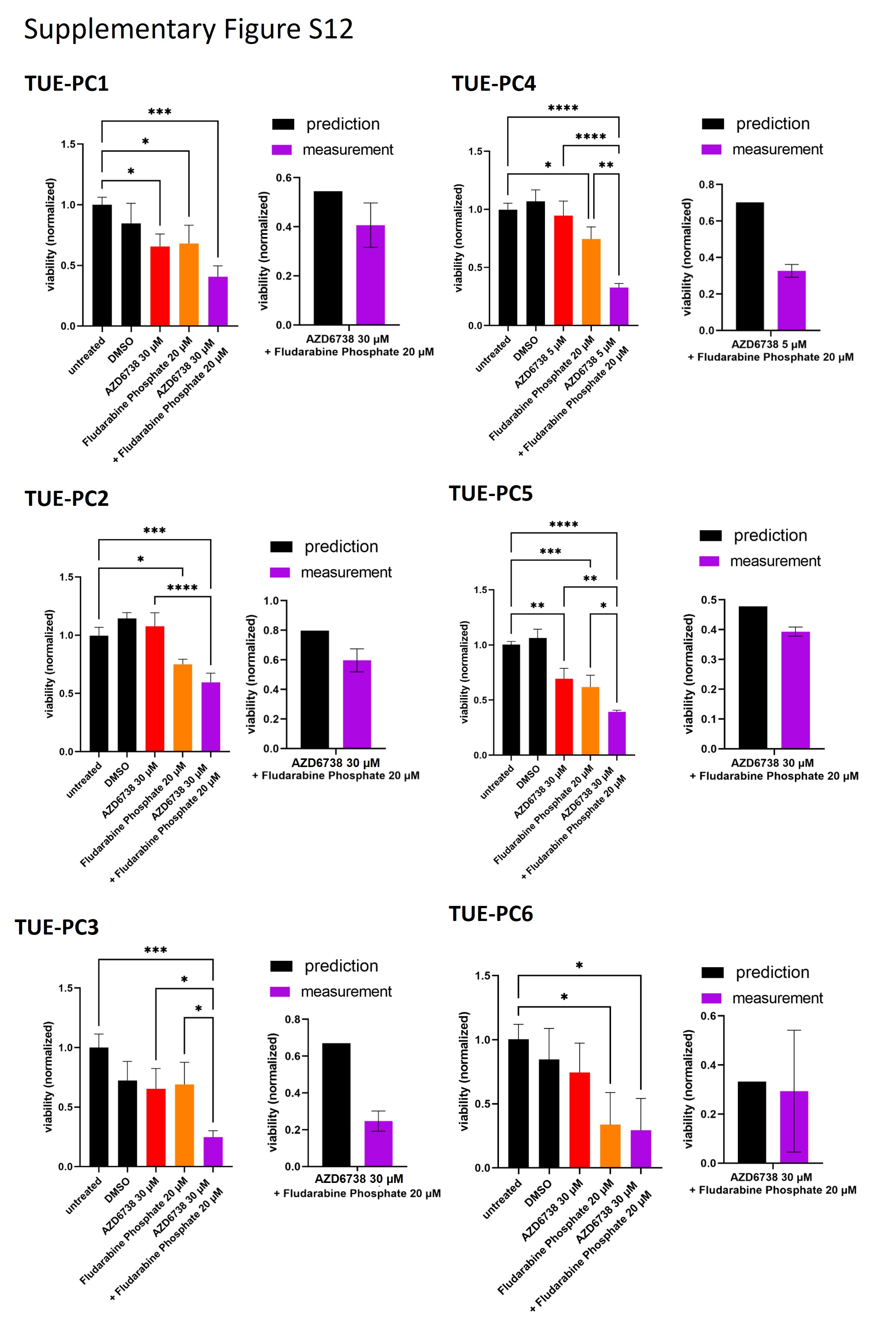

Supplement: Supplementary file 12 — Supplementary Material 12 [file 13046_2024_2995_MOESM12_ESM.jpg]

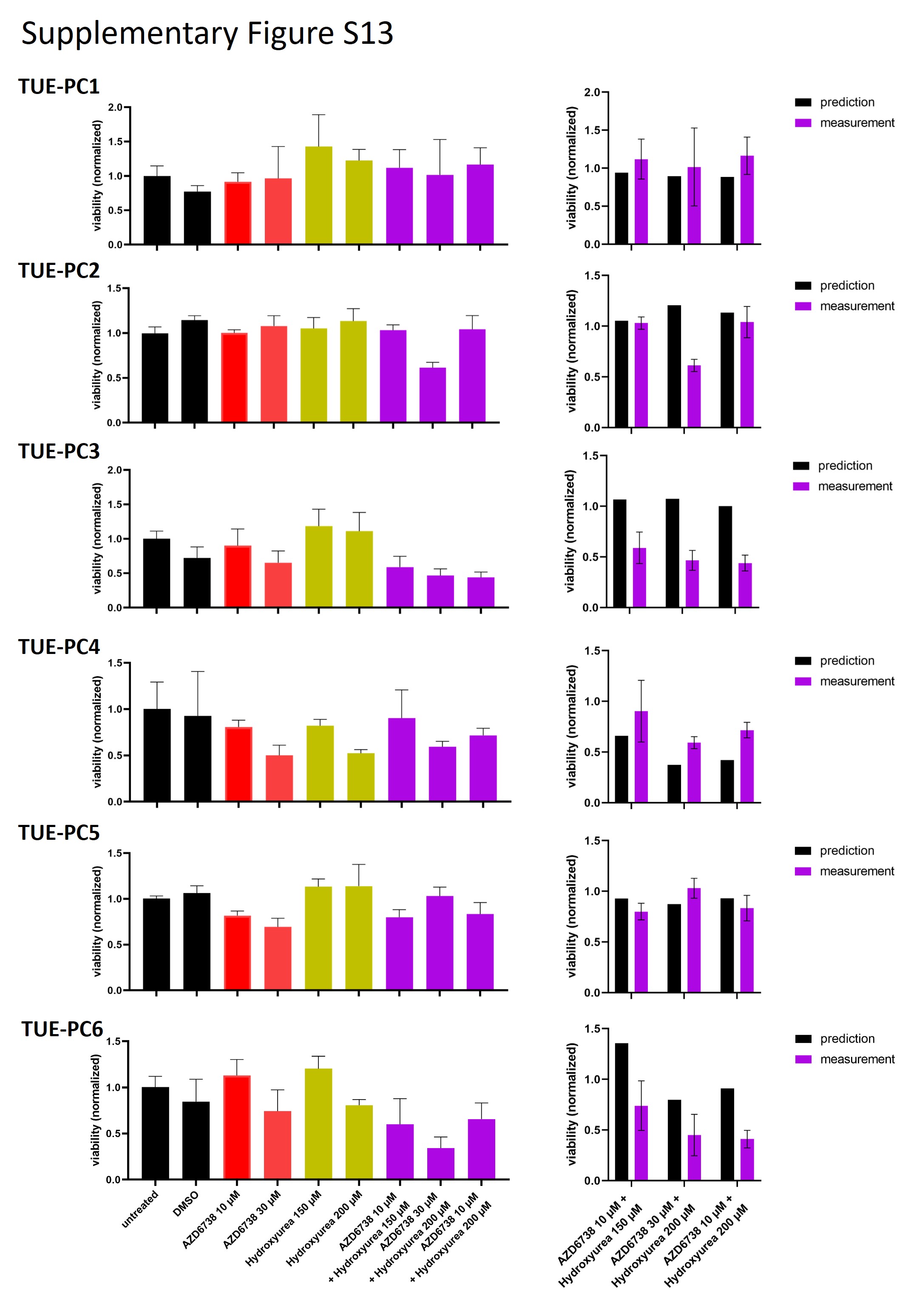

Supplement: Supplementary file 13 — Supplementary Material 13 [file 13046_2024_2995_MOESM13_ESM.jpg]
