# Supplementary material for: Functionally-instructed modifiers of response to ATR inhibition in experimental glioma
Source: J Exp Clin Cancer Res. 2024 Mar 12;43:77. doi: 10.1186/s13046-024-02995-z (PMC10935927; doi:10.1186/s13046-024-02995-z)
Supplement: Supplementary file 14 — Supplementary Material 14 [file 13046_2024_2995_MOESM14_ESM.docx]

**Supplementary Table ST1: Scoring Sheet**

| **1** | **general appearance** | |  |
| --- | --- | --- | --- |
| **a** | state of care | clean, shiny, smooth fur | 0 |
|  |  | no fur grooming, dull fur | 1 |
|  |  | no fur grooming, dirty | 2 |
|  |  | no fur grooming, dirty, piloerection | 4 |
| **b** | Eyes | Normal | 0 |
|  |  | subtly sunk in, swollen | 1 |
|  |  | lids closed | 2 |
|  |  | strongly sunken in, lids closed, sticky | 4 |
| **c** | Posture | Normal | 0 |
|  |  | slightly bent | 1 |
|  |  | strongly bent | 2 |
|  |  | strongly bent, paws under the body | 4 |
| **d** | Breathing | regular | 0 |
|  |  | regular, slightly enhanced | 1 |
|  |  | strongly enhanced | 2 |
|  |  | difficulty breathing, pumping | 4 |
| **e** | behavior/activity | normal | 0 |
|  |  | slightly changed | 1 |
|  |  | reduced spontaneous-explorative behavior, isolated | 2 |
|  |  | apathic, inactivity | 4 |
| **2** | **nutritional status** | |  |
| **a** | Bodycondition score | vertebrae and pelvic bones only palpable when slightly pressed | 0 |
|  |  | vertebrae and pelvic bones easily palpable, abdominal retraction detectable from the side | 1 |
|  |  | vertebrae visible, pelvic bones palpable | 2 |
|  |  | vertebrae, pelvic bones and rips visible | 4 |
| **b** | Weight | normal, continuous increase (±5%) | 0 |
|  | calculated from original weight, corrected for expected weight gain of healthy animals | weight loss 5-10% | 1 |
|  |  | weight loss >10 bis <20% | 2 |
|  |  | weight loss max. 20% | **end point** |
| **3** | **experiment associated: tumor growth** | |  |
| **a** | Neurological symptoms: | none | 0 |
|  | pen test, grid, lef paw paralysis | slight loss-of-balance, occasionally missed steps, slight paralysis | 1 |
|  |  | Moderate loss-of-balance, every third step missed, moderate paralysis | 2 |
|  |  | Strong loss-of-balance, total inactivity, strong paralysis | **end point** |
| **b** | Grimace Scale | normal | 0 |
|  |  | 1 pain score | 2 |
|  |  | More than 1 pain score | **end point** |
| **end points** | 0 -2 points | Normal | |
|  | 3 -8 points | daily scoring/weight control, might talk to veterinarians (pain killers, wet food) | |
|  | 9 and more points | **end point** | |
|  | 4 points in experiment associated criteria | **end point** | |
| **exceptional measures** | weight loss 20% | | |
|  | neurological symptoms with strong loss-of-balance, total inactivity and strong paralysis | | |
|  | More than 1 pain score | | |
|  | 1x Score 4 | | |

**Supplementary Table ST2: Antibody list for DigiWest**

| **Antigen** | **Mod-Site** | **Supplier** | **Product No.** | **Species** | **MW (kDa)** |
| --- | --- | --- | --- | --- | --- |
| 14-3-3 sigma |  | R&D | AF4424 | gt | 28 |
| 53BP1 |  | Cell Signaling | 4937 | rb | 450 |
| 53BP1 – phospho | Thr543 | Cell Signaling | 3428 | rb | 450 |
| Akt |  | Cell Signaling | 4685 | rb | 60 |
| Akt – phospho | Ser473 | Cell Signaling | 4060 | rb | 60 |
| Akt – phospho | Thr308 | Cell Signaling | 13038 | rb | 60 |
| Apaf-1 |  | Cell Signaling | 8723 | rb | 135 |
| ATM |  | Cell Signaling | 2873 | rb | 350 |
| ATM – phospho | Ser1981 | Cell Signaling | 5883 | rb | 350 |
| ATR |  | Cell Signaling | 2790 | rb | 250 |
| ATR - phospho | Ser428 | Cell Signaling | 2853 | rb | 300 |
| Aurora B (AIM1) |  | Cell Signaling | 3094 | rb | 40 |
| Aven |  | Cell Signaling | 2300 | rb | 50 |
| Bad |  | Cell Signaling | 9239 | rb | 23 |
| Bad - phospho | Ser136 | Cell Signaling | 4366 | rb | 23 |
| Bax |  | Cell Signaling | 2772 | rb | 20 |
| Bcl2 |  | Cell Signaling | 4223 | rb | 26 |
| Bcl2 - phospho | Ser70 | Cell Signaling | 2827 | rb | 28 |
| Bcl-xL |  | Cell Signaling | 2764 | rb | 30 |
| BID |  | Cell Signaling | 2006 | ms | 22 |
| BRCA1 |  | Cell Signaling | 14823 | rb | 220 |
| Caspase 3 |  | Cell Signaling | 9662 | rb | 35, 19, 17 |
| Caspase 3 - cleaved | Asp175 | Cell Signaling | 9661 | rb | 19, 17 |
| Caspase 7 |  | Cell Signaling | 9492 | rb | 35, 20 |
| Caspase 8 |  | Cell Signaling | 9746 | ms | 43, 18 |
| Caspase 9 |  | Cell Signaling | 9502 | rb | 35, 17 |
| Caspase 9 - phospho | Ser196 | Thermo Fisher | PA5-40222 | rb | 46 |
| CBP |  | Cell Signaling | 7389 | rb | 300 |
| cdc2 (CDK1) |  | Cell Signaling | 9112 | rb | 34 |
| cdc2 (CDK1) - phospho | Tyr15 | Cell Signaling | 4539 | rb | 34 |
| cdc25A |  | abm | Y021163 | rb | 59 |
| cdc25A - phospho | Ser75 | abm | Y011138 | rb | 59 |
| cdc25C |  | Epitomics | 1302-1 | rb | 60 |
| CDK2 |  | Cell Signaling | 2546 | rb | 33 |
| CDK2 - phospho | Thr160 | Cell Signaling | 2561 | rb | 33 |
| CDK4 |  | Cell Signaling | 12790 | rb | 30 |
| CDK5 |  | Cell Signaling | 2506 | rb | 30 |
| CDK6 |  | Santa Cruz | sc-7961 | ms | 40 |
| CDK6 - phospho | Tyr13 | biorbyt | orb15013 | rb | 36 |
| CDK6 - phospho | Tyr24 | biorbyt | orb15014 | rb | 36 |
| CDKN2A |  | ProteinTech Group | 10883-1-AP | rb | 17 |
| CDKN2B |  | Bio-Techne | MAB6798 | ms | 15 |
| Chk1 - phospho | Ser345 | Cell Signaling | 2341 | rb | 56 |
| Chk1 - phospho | Ser296 | Cell Signaling | 2349 | rb | 56 |
| Chk1 - phospho | Ser296 | Cell Signaling | 2349 | rb | 56 |
| Chk2 |  | Cell Signaling | 3440 | ms | 62 |
| Chk2 - phospho | Thr68 | Cell Signaling | 2661 | rb | 62 |
| CHOP |  | Cell Signaling | 2895 | ms | 27 |
| c-Jun |  | Cell Signaling | 9165 | rb | 48, 43 |
| c-Jun - phospho | Ser73 | Cell Signaling | 3270 | rb | 48 |
| c-myc |  | Cell Signaling | 9402 | rb | 70-57 |
| c-myc - phospho | Thr58/Ser62 | abcam (Epitomics) | ab32029 (1203-1) | rb | 57 |
| c-myc - phospho | Thr58 | Thermo Fisher | PA5-37654 | rb | 62 |
| Cyclin A |  | abcam | ab53054 | rb | 49 |
| Cyclin B1 |  | abcam | ab32053 | rb | 58 |
| Cyclin D1 |  | Cell Signaling | 2926 | ms | 36 |
| Cyclin D2 |  | Cell Signaling | 3741 | rb | 31 |
| Cyclin E1 |  | Cell Signaling | 4129 | ms | 48 |
| Cytochrome c |  | Cell Signaling | 4280 | rb | 14 |
| DNA polymerase beta |  | abcam (Epitomics) | ab175197 (8220-1) | rb | 38 |
| DNA-PK |  | Cell Signaling | 4602 | rb | 450 |
| EGFR (ErB-1, HER1) |  | Cell Signaling | 4267 | rb | 175 |
| EGFR (ErB-1, HER1) - phospho | Tyr1068 | Cell Signaling | 2234 | rb | 175 |
| EMSY |  | abcam (Epitomics) | ab32329 (1602-1) | rb | 141 |
| Erk1/2 (MAPK p44/42) |  | Cell Signaling | 4695 | rb | 44, 42 |
| Erk1/2 (MAPK p44/42) - phospho | Thr202/Tyr204 | Cell Signaling | 9101 | rb | 44, 42 |
| Ezh2 |  | Cell Signaling | 5246S | rb | 98 |
| FAK1 |  | Cell Signaling | 3285 | rb | 125 |
| FAK1 - phospho | Tyr397 | Cell Signaling | 8556 | rb | 125 |
| FAS |  | Cell Signaling | 4233 | rb | 50-40 |
| FasL |  | Cell Signaling | 4273 | rb | 40, 26 |
| FGF receptor 1 |  | Cell Signaling | 9740 | rb | 145, 120, 92 |
| GADD45 alpha |  | Cell Signaling | 4632 | rb | 22 |
| GADD45B |  | abcam (Epitomics) | ab128920 (5833-1) | rb | 18 |
| GSK3 beta |  | Cell Signaling | 9315 | rb | 46 |
| GSK3 beta - phospho | Ser9 | Cell Signaling | 9336 | rb | 46 |
| Histone deacetylase 1 (HDAC1) |  | Cell Signaling | 2062 | rb | 62 |
| Histone deacetylase 2 (HDAC2) |  | Epitomics | 1603-1 | rb | 55 |
| Histone H2A.X - phospho | Ser139 | Cell Signaling | 9718 | rb | 15 |
| Histone H3 - acetyl | Lys9/Lys14 | Calbiochem | 382158 | rb | 17 |
| Histone H3 - monomethyl | Lys4 | Cell Signaling | 5326 | rb | 17 |
| Histone H3 - phospho | Ser10 | Cell Signaling | 9701 | rb | 17 |
| Histone H3 - trimethyl | Lys27 | Cell Signaling | 9756 | rb | 17 |
| HSF1 |  | Epitomics | 2043-1 | rb | 82 |
| HSF1 - phospho | Ser326 | Epitomics | 2092-1 | rb | 82 |
| IGFBP-3 |  | abcam | ab137370 | rb | 32 |
| IkappaB alpha |  | Cell Signaling | 9242 | rb | 41 |
| IkappaB alpha |  | Cell Signaling | 9242 | rb | 41 |
| IkappaB alpha - phospho | Ser32 | Cell Signaling | 9241 | rb | 41 |
| IKK alpha |  | Cell Signaling | 2682 | rb | 85 |
| IKK alpha/beta - phospho | Ser176/177 | Cell Signaling | 2078 | rb | 87, 85 |
| IKK beta |  | Cell Signaling | 2370 | rb | 87 |
| IKK epsilon |  | Cell Signaling | 2905 | rb | 80 |
| IL-6 |  | Cell Signaling | 12153 | rb | 21-28 |
| IL-8 |  | Cell Signaling | 94407 | rb | 11 |
| Jak 2 |  | Cell Signaling | 3229 | rb | 125 |
| Jak 2 - phospho | Tyr1007/Tyr1008 | Cell Signaling | 3771 | rb | 125 |
| JNK/SAPK |  | Cell Signaling | 9252 | rb | 54, 46 |
| JNK/SAPK 1/2/3 - phospho | Thr183/Tyr185 | Santa Cruz | sc-6254 | ms | 54, 46 |
| Ku80 |  | Cell Signaling | 2180 | rb | 86 |
| Mcl-1 |  | Cell Signaling | 5453 | rb | 40, 35 |
| MDM2 |  | Santa Cruz | sc-965 | ms | 90, 60 |
| MDM2 - phospho | Ser166 | Life Technologies | 44-1400G | rb | 125 |
| MEK1/2 |  | Cell Signaling | 9126 | rb | 45 |
| MKK4 (SEK1) - phospho | Ser257/Thr261 | Cell Signaling | 9156 | rb | 44 |
| MMP13 |  | R&D | MAB511 | ms | 54 |
| MMP7 |  | R&D | MAB9071 | ms | 30 |
| MMP-9 |  | Cell Signaling | 13667 | rb | 92, 84 |
| Mre11 |  | Cell Signaling | 4847 | rb | 81 |
| Mre11 – phospho | Ser676 | Cell Signaling | 4859 | rb | 81 |
| mTOR (FRAP) |  | Cell Signaling | 2983 | rb | 289 |
| mTOR (FRAP)- phospho | Ser2448 | Cell Signaling | 5536 | rb | 289 |
| NF-κB p100/p52 |  | Cell Signaling | 4882 | rb | 120, 52 |
| NF-κB p105/p50 |  | Cell Signaling | 3035 | rb | 120, 50 |
| NF-κB p65 |  | Epitomics | 2229-1 | rb | 70 |
| NF-κB p65 – phospho | Ser468 | Cell Signaling | 3039 | rb | 65 |
| P21 – phospho | Thr145 | Invitrogen | PA512646 | rb | 18 |
| p21 (Waf1, Cip1, CDKN1A) |  | Cell Signaling | 2947 | rb | 21 |
| p27 (Kip1, CDKN1B) |  | Cell Signaling | 3698 | ms | 27 |
| p27 (Kip1, CDKN1B) – phospho | Ser10 | abcam (Epitomics) | ab62364 | rb | 22 |
| p53 |  | R&D | af1355 | gt | 53 |
| p53 – acetyl | Lys305 | abcam (Epitomics) | ab109396 (3308-1) | rb | 44 |
| p53 – phospho | Ser15 | Cell Signaling | 9284 | rb | 53 |
| p53 – phospho | Ser20 | Cell Signaling | 9287 | rb | 53 |
| p53 – phospho | Ser37 | Cell Signaling | 9289 | rb | 53 |
| p95 (NBS1) – phospho | Ser343 | Cell Signaling | 3001 | rb | 95 |
| PAI-1 |  | Cell Signaling | 11907 | rb | 48 |
| PARP |  | Cell Signaling | 9532 | rb | 116, 89 |
| PARP – cleaved | Asp214 | Cell Signaling | 9541 | rb | 89 |
| PI3-kinase p110 alpha |  | Cell Signaling | 4255 | rb | 110 |
| PI3-kinase p110 beta |  | Millipore | 04-400 | rb | 110 |
| PI3-kinase p85 alpha |  | abcam (Epitomics) | ab40755 (1675-1) | rb | 85 |
| PI3-kinase p85/p55 – phosphor | Tyr458/Tyr199 | Cell Signaling | 4228 | rb | 85, 60 |
| PKR |  | Cell Signaling | 2766 | rb | 67 |
| PKR – phospho | Thr446 | abcam | ab47377-100 | rb | 62 |
| PTEN |  | Cell Signaling | 9552 | rb | 54 |
| PTEN – phospho | Ser380 | Cell Signaling | 9551 | rb | 54 |
| Rad51 |  | Epitomics | 3161-1 | rb | 37 |
| Rb |  | Cell Signaling | 9313 | rb | 110 |
| Rb – phospho | Ser780 | Cell Signaling | 3590 | rb | 110 |
| Rb – phospho | Ser807/Ser811 | Cell Signaling | 8516 | rb | 110 |
| Rb2 (p130) |  | abcam (Epitomics) | ab76234 (2130-1) | rb | 128 |
| Rb2 (p130) – phospho | Ser952 | abcam (Epitomics) | ab68136 (2272-1) | rb | 128 |
| RecQL1 |  | Santa Cruz | sc-25547 | rb | 75 |
| Rictor |  | Cell Signaling | 2114 | rb | 200 |
| SMC1 – phospho | Ser957 | Cell Signaling | 4805 | ms | 145 |
| STAT 3 |  | Cell Signaling | 4904 | rb | 86, 79 |
| STAT 3 – phospho | Tyr705 | Cell Signaling | 9145 | rb | 86, 79 |
| STAT 3 – phospho | Ser727 | Cell Signaling | 9134 | rb | 86 |
| Survivin |  | Cell Signaling | 2802 | ms | 16 |
| Survivin – phospho | Thr34 | Cell Signaling | 8888 | rb | 18-16 |
| TAK1 |  | Cell Signaling | 4505 | rb | 82-78 |
| VEGF-A |  | Dako | M7273 | ms | 45 |
| XIAP |  | Santa Cruz | sc-55550 | ms | 55 |
| XLF |  | Cell Signaling | 2854 | rb | 39 |

***Abbreviations:*** *Asp, asparagine; gt, goat; Lys, lysine; ms, mouse; rb, rabbit; Ser, serine; Thr, threonine; Tyr, tyrosine;* genes (alphabetical order): *53BP1, p53-binding protein 1; Akt, AKT serine/threonine kinase 1; Apaf, apoptotic peptidase activating factor 1; ATM, ATM serine/threonine kinase; ATR, ataxia telangiectasia and Rad3 related serine/threonine kinase; AIM, absent in melanoma; Bad, Bcl-2 associated agonist of dell ceath protein; Bax, Bcl-2 associated X protein; Bcl-2, B-cell lymphoma 2; BID, BH3-interacting domain death agonist; BRCA1, breast cancer type 1 susceptibility protein; CBP, CREB-binding protein; cdc, cell division control; CDK, cyclin-dependent kinase; CDKN2A, cyclin-dependent kinase inhibitor 2A; Chk, checkpoint kinase; CHOP, C/EBP homologous protein; DNA-PK, DNA-dependent protein kinase; EGFR, epidermal growth factor receptor; Erk, extracellular-signal regulated kinase; Ezh2, enhancer of zeste homolog 2; FAK, focal adhesion kinase; FGF, fibroblast growth factor; GADD45, DNA-damage-inducible protein; GSK, glycogen synthase kinase; HSF1, heat shock factor 1; IGFBP, insulin-like growth factor binding proteins; IKK, IkappaB kinase; IL, interleukine; Jak, Janus kinase; JNK/SAPK, jun amino-terminal kinase/stress-activated protein kinase; Mcl-1, induced myeloid leukemia cell differentiation protein; MDM2, mouse couble minute 2 homolog; MEK/MKK, mitogen-activated protein kinase kinase; MMP, matrix metalloproteinase; Mre, double-strand break repair protein; mTOR, mammalian target of rapamycin; FRAP, FKBP-12-rapamycin associated protein; NF-κB, nuclear factor ‘kappa-light-chain-enhancer’ of activated B-cells; NBS1, nibrin; PAI-1, plasminogen activator inhibitor-1; PARP, Poly(ADP-ribose)polymerase; PI3-kinase, phosphoinositide 3-kinase; PKR, protein kinase RNA-activated; PTEN, phosphatase and tensin homolog; RAD51, DNA repair protein RAD51 homolog 1; Rb, retinoblastoma; RecQL1; RecQ Like Helicase; Rictor, Rapamycin-insensitive companion of mammalian target of rapamycin; SMC1, structural maintenance of chromosomes protein 1; STAT 3; signal transducer and activator of transcription 3; TAK1, transforming growth factor beta-activated kinase 1; VEGF, vascular endothelial growth factor; XIAP, X-linked inhibitor of apoptosis; XLF, XRCC4-like factor.*

**Supplementary Table ST3: Compound list**

| **Compound** | **Supplier** | **Stock concentration** | **Diluent** | **Description** | **Evidence: genetic vulnerability in screen/ …** |
| --- | --- | --- | --- | --- | --- |
| AZD6738 | Selleckchem (Houston, TX, US) | 50 mM | DMSO | ATR inhibitor | n/a |
| Berzosertib | ChemiTek (Indianapolis, IN, US) | 50 mM | DMSO | ATR inhibitor | n/a |
| AZD5438 | Selleckchem (Houston, TX, US) | 10 mM | DMSO | Multiple CDK inhibitor | CDK7, CDK12; cell cycle analyses |
| Dinaciclib | Selleckchem (Houston, TX, US) | 10 mM | DMSO | Multiple CDK inhibitor | CDK7, CDK12; cell cycle analyses |
| Tideglusib | Selleckchem (Houston, TX, US) | 10 mM | DMSO | GSK3B inhibitor | GSK3B |
| AZD1080 | Selleckchem (Houston, TX, US) | 10 mM | DMSO | GSK3B inhibitor | GSK3B |
| Harmine | Selleckchem (Houston, TX, US) | 10 mM | DMSO | DYRK1A inhibitor | DYRK1A |
| Bortezomib | Selleckchem (Houston, TX, US) | 10 mM | DMSO | 26S proteasome inhibitor | PSMD3 |
| Doxorubicin | Selleckchem (Houston, TX, US) | 10 mM | DMSO | Chemotherapeutic | FANC, TOP2A |
| Argyrin F | kindly provided by Prof. N. Malek (Tübingen, DE) and Prof. M Kalesse (Hannover, DE) | 5 mg/mL | DMSO | 20S proteasome inhibitor | PSMD3 |
| Everolimus | Selleckchem (Houston, TX, US) | 10 mM | DMSO | mTOR inhibitor | RNASeq |
| Hydroxyurea | MedChemExpress (Monmouth Junction, NJ, US) | 100 mM | H2O | RRM1/2 inhibitor | RRM1/RRM2 |
| Olaparib | Selleckchem (Houston, TX, US) | 10 mM | DMSO | PARP1 inhibitor | BRCA2 |
| Paxalisib | Selleckchem (Houston, TX, US) | 10 mM | DMSO | PI3K/mTOR inhibitor | RNASeq |
| Temozolomide | Excella Pharmasource (Feucht, DE) | 100 mM | DMSO | Chemotherapeutic | Standard of care |
| Vorinostat | MedChemExpress (Monmouth Junction, NJ, US) | 10 mM | DMSO | HDAC inhibitor | BIRC5 (Survivin); RNASeq |
| Daprodustat | MedChemExpress (Monmouth Junction, NJ, US) | 10 mM | DMSO | HIF inhibitor | EGLN1 |
| Cisplatin | MedChemExpress (Monmouth Junction, NJ, US) | 1 mM | H2O | Chemotherapeutic | FANC |
| Cyclosporin | MedChemExpress (Monmouth Junction, NJ, US) | 10 mM | DMSO | Immunosuppressant | ABCG2 |
| Etoposide | MedChemExpress (Monmouth Junction, NJ, US) | 10 mM | DMSO | Chemotherapeutic | TOP2A |
| Febuxostat | MedChemExpress (Monmouth Junction, NJ, US) | 10 mM | DMSO | ABCG2 inhibitor | ABCG2 |
| Fludarabine Phosphate | MedChemExpress (Monmouth Junction, NJ, US) | 10 mM | DMSO | Chemotherapeutic, RRM1/2, POLE, POLA1, POLD1 inhibitor | RRM1/RRM2, POLE3 |
| Fluorouracil | MedChemExpress (Monmouth Junction, NJ, US) | 10 mM | H2O | Chemotherapeutic, TYMS, DTYMK inhibitor | TYMS, DTYMK |
| Gemcitabine | MedChemExpress (Monmouth Junction, NJ, US) | 100 mM | DMSO | Chemotherapeutic | RRM1/RRM2, FANC |
| Lenalidomide | MedChemExpress (Monmouth Junction, NJ, US) | 100 mM | DMSO | target E3 ubiquitin ligase | DDB1 |
| Mitoxantrone | MedChemExpress (Monmouth Junction, NJ, US) | 10 mM | DMSO | Antibiotic, topoisomerase inhibitor | TOP2A |
| Niraparib | MedChemExpress (Monmouth Junction, NJ, US) | 10 mM | DMSO | PARP inhibitor | BRCA2 |
| THZ1 | MedChemExpress (Monmouth Junction, NJ, US) | 10 mM | DMSO | CDK7 (also CDK12/13) inhibitor | CDK7, CDK12; cell cycle analyses |
| Voacamine | MedChemExpress (Monmouth Junction, NJ, US) | 10 mM | DMSO | CB1 antagonist, ABCB1 inhibitor | ABCB1 |
| Zotiraciclib | MedChemExpress (Monmouth Junction, NJ, US) | 10 mM | DMSO | Multiple CDK inhibitor | CDK12, CDK13; cell cycle analyses |

***Abbreviations****: ABCB1, ATP binding cassette subfamily B member 1; ABCG2, ATP binding cassette subfamily G member 2; ATR, ataxia telangiectasia and Rad3 related; BIRC5, baculoviral inhibitor of apoptosis repeat-containing 5; BRCA2, breast cancer type 2 susceptibility protein; CB1, cannabinoid-receptor type 1; CDK, cyclin-dependent kinase; DDB1, DNA damage-binding protein 1; DMSO, dimethyl sulfoxide; DTYMK, deoxythymidylate kinase; DYRK1A, dual-specificity tyrosine phosphorylation-regulated kinase 1A; EGLN1, egl-9 family hypoxia inducible factor 1; FANC, fanconi anemia complementation group; GSK3B, glycogen synthase kinase-3 beta; HDAC, histone deacetylase; HIF, hypoxia-inducible factor; mTOR, mammalian target of rapamycin; n/a, not available; PARP, poly(ADP-ribose)polymerase; PI3K, posphoinositide 3-kinase; POLA1, DNA polymerase alpha 1; POLD1, DNA polymerase delta; POLE, DNA polymerase epsilon; PSMD3, proteasome 26S subunit, non-ATPase 3; RRM1/2, ribonucleotide reductase catalytic subunit M1/M2; TOP2A, DNA topoisomerase II alpha; TYMS, thymidylate synthase*

**Supplementary Table ST4: Basic characteristics of primary cultures**

| **ID** | **Diagnosis** | **IDH status** | **MGMT gene promoter** |
| --- | --- | --- | --- |
| TUE-PC1 | Glioblastoma | Wildtype | Unmethylated |
| TUE-PC2 | Glioblastoma | Wildtype | Methylated |
| TUE-PC3 | Glioblastoma | Wildtype | Unmethylated |
| TUE-PC4 | Metastasis (Adenocarcinoma) | n/a | n/a |
| TUE-PC5 | Glioblastoma | Wildtype | Unmethylated |
| TUE-PC6 | Glioblastoma | Wildtype | Unmethylated |

***Abbreviations****: IDH, isocitrate dehydrogenase; MGMT, O6-methylguanine-DNA methyltransferase; PC, primary glioma culture*
